# Mathematical models for cytarabine-derived myelosuppression in acute myeloid leukaemia

**DOI:** 10.1371/journal.pone.0204540

**Published:** 2019-07-01

**Authors:** Felix Jost, Enrico Schalk, Kristine Rinke, Thomas Fischer, Sebastian Sager

**Affiliations:** 1 Institute of Mathematical Optimization, Faculty of Mathematics, Otto-von-Guericke University, Magdeburg, Germany; 2 Department of Hematology and Oncology, University Medical Center, Otto-von-Guericke-University, Magdeburg, Germany; German Cancer Research Center (DKFZ), GERMANY

## Abstract

We investigate the personalisation and prediction accuracy of mathematical models for white blood cell (WBC) count dynamics during consolidation treatment using intermediate or high-dose cytarabine (Ara-C) in acute myeloid leukaemia (AML). Ara-C is the clinically most relevant cytotoxic agent for AML treatment. We extend a mathematical model of myelosuppression and a pharmacokinetic model of Ara-C with different hypotheses of Ara-C’s pharmacodynamic effects. We cross-validate the 12 model variations using dense WBC count measurements from 23 AML patients. Surprisingly, the prediction accuracy remains satisfactory in each of the models despite different modelling hypotheses. Therefore, we compare average clinical and calculated WBC recovery times for different Ara-C schedules as a successful methodology for model discrimination. As a result, a new hypothesis of a secondary pharmacodynamic effect on the proliferation rate seems plausible. Furthermore, we demonstrate the impact of treatment timing on subsequent nadir values based on personalised predictions as a possibility for influencing/controlling myelosuppression.

## Introduction

Acute myeloid leukaemia (AML) is a malignant clonal disorder of myeloid stem and progenitor cells. In untreated AML, immature neoplastic myeloid blasts rapidly proliferate and suppress the generation and maturation of blood cells in the bone marrow. While being a curable disease using chemotherapy including anthracyclines and/or cytarabine (Ara-C), this approach leads to prolonged myelosuppression with extremely low white blood cell (WBC) counts (leukopenia), i.e. values below 1 *G*/*L*, associated with a high risk of infection and treatment-related mortality [1].

Consolidation treatment, repetitive (up to 4) cycles of intermediate-/high-dose Ara-C (1 − 3 *g*/*m*^2^) [2], is given once patients achieve complete remission (CR) and is considered the most important part of chemotherapy in preventing relapses. The treatment of 3 *g*/*m*^2^ Ara-C infusion lasting 3 hours every 12 hours on days 1, 3 and 5 for patients aged 60 years and younger was established by Mayer *et al*. [3] and remains the current standard of treatment to this day.

If predictions from personalised mathematical models including all relevant biomarkers were reliable and accurate, they could be used for providing better care to AML patients receiving Ara-C consolidation treatment, e.g. in an automatised measurement–decision support loop [4, 5]. Precisely identifying the period of Ara-C-induced profound leukopenia and modification of treatment schedules based on such predictions might enable prevention of severe infectious complications, sepsis, and thus delay to undergo subsequent treatment cycles. Therefore, by realising timely adherence to consolidation therapy cycles and by avoiding delays in treatment schedule, the density of chemotherapy cycles may be increased and thus deeper remissions and lower relapse rates may be achieved. This may ultimately translate into improved overall survival rates.

There are many different levels on which haematopoiesis [6–8], granulopoiesis [9–11], myelosuppression [12] and dynamics of leukemic cells [8] can be modelled [13, 14]. A comprehensive overview and summary of the various models is given in the recently published reviews [15, 16]. We analysed models that capture only the most important dynamics for non-leukemic cells and “agglomerate” different physiological effects into simplified expressions. Higher levels of detail in more sophisticated models, covering many physiologial properties and thus providing a deeper understanding of biological phenomena, come at the price of needing more observed biomarkers and model parameters. Depending on the medical question, the required outcome and the available biomarker information, more complex models can be reformulated into minimalistic models that concentrate on the fundamental physiological mechanisms without a qualitative loss of the outcome [17, 18]. In our setting, the current lack of clinical measurements of endogenous granulocyte-colony stimulating factor (G-CSF) concentrations and leukemic cell counts (as no relapse events occurred) leads to identifiability issues with the related dynamics. Due to these issues and our main focus on myelosuppression and WBC recovery, we concentrated on agglomerating effects of Ara-C on proliferation and maturation rates and did not consider models including G-CSF or leukemic cell dynamics.

Mathematical models for myelosuppression due to various chemotherapy agents have been proposed [12, 19–23] and applied successfully to predict the dynamics of neutrophils [4, 24]. However, this is not the fact for high-dose Ara-C, the most important component in consolidation therapy [2, 25]. Pharmacology of Ara-C is particularly difficult, as its exact mechanisms of action both on normal and leukemic cells are not fully understood. The main effect of Ara-C on normal and leukemic proliferating cells is the inclusion of intracellular Ara-C triphosphate (Ara-CTP) into DNA and RNA, which impairs cell replication [26]. Yet, the synthesis of intracellular Ara-CTP is saturable such that the clinical success of intermediate-/high-dose Ara-C is not well explained [3, 27, 28]. Additional effects are the subject of ongoing research [28, 29].

Here, we surveyed different published and new hypotheses of the pharmacodynamic (PD) effects of Ara-C on WBC dynamics during AML consolidation therapy. We used models for myelosuppression and Ara-C pharmacokinetics (PK) from the literature to quantify prediction accuracies. All model variations are based on the myelosuppression model developed by Friberg *et al*. [12] and are tailored to the special case of Ara-C via a parameterised two-compartment PK model. The general modelling goals were to include possible secondary effects of Ara-C and to obtain a good balance between modelling detail, prediction accuracy, and the number of patient-specific parameters. As a successful methodology, we considered predictions of WBC recovery times *t*_rec_ (defined as the time when the WBC count recovers above 1 *G*/*L*) for *different* Ara-C schedules and compared them to published average WBC recovery times.

Our work is another contribution towards the ultimate goal of mathematically optimising and individualising consolidation therapy for AML patients. Here, we are focusing on one important aspect of AML therapy: Ara-C-derived myelosuppression.

### Remark on terminology and potentially confusing synonyms

The manuscript is categorised in the intersection of mathematics, control theory, systems biology, pharmacology, and medicine. Words like “model” or “parameter” have different meanings in these scientific communities, and similar concepts have different names like “calibration”, “estimation”, or “personalisation”. For convenience, we list some synonyms that we did (not) use in S1 Table.

## Methods

The section starts with a detailed description of the different model variations. Next, three initial value approaches are introduced which are used during parameter estimation. Afterwards we describe our clinical data and specify how we personalised the mathematical models. The sections *Prediction & Cross-Validation* and *Schedule Timing* contain the description of several simulation studies which were performed to discriminate between the different model variations and to analyse the treatment timing in consecutive CCs on the nadir value. The mathematical approaches to parameter estimation, uncertainty quantification, statistical analysis and the nonlinear mixed-effects modelling approach can be found in the S1 Appendix.

### Mathematical Models

**Fig 1:**
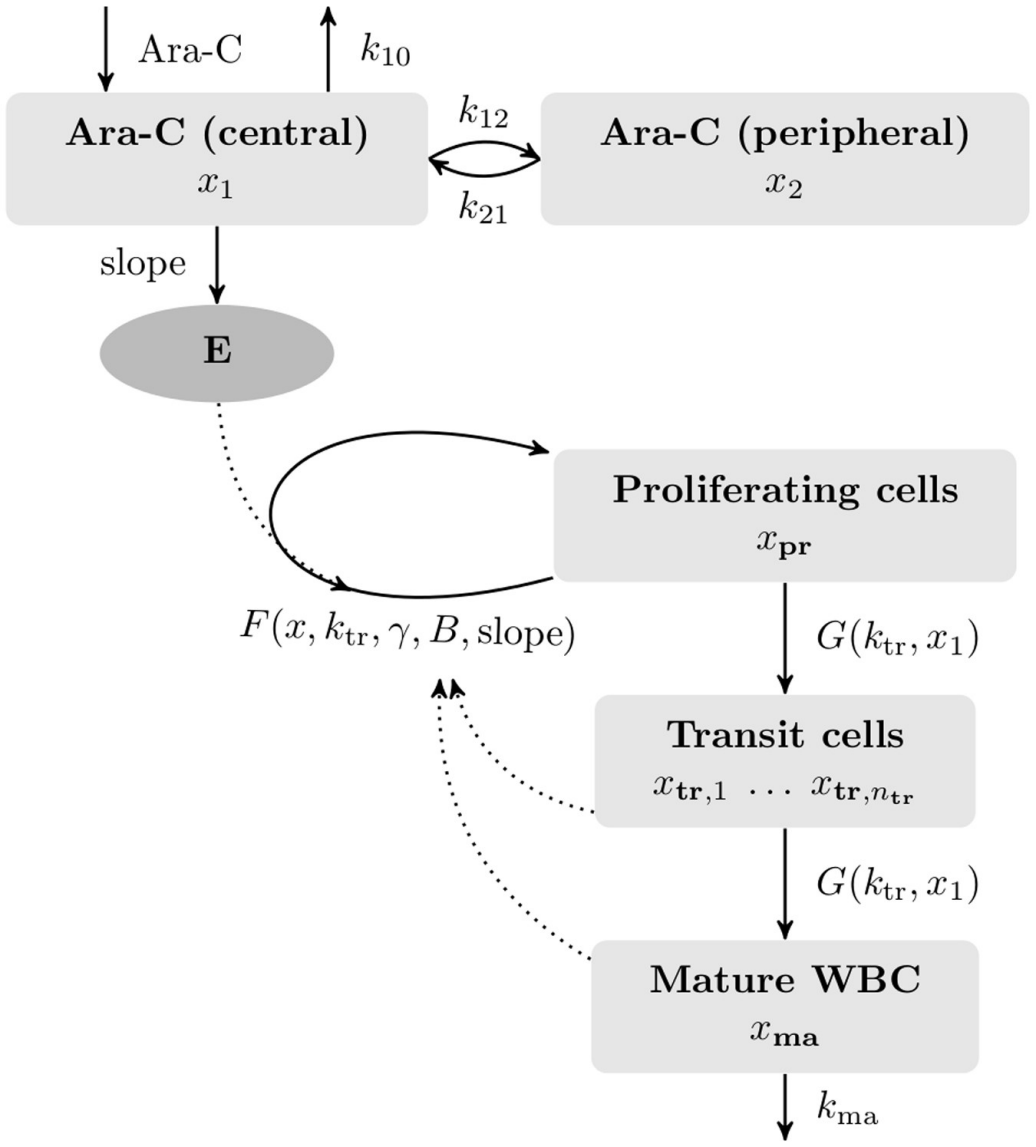
Schematic model from which all mathematical models were derived. We assumed clustering of cells and cytarabine (Ara-C) concentrations in compartments with identical properties. White blood cell (WBC) differentiation is represented by a proliferating compartment *x*_pr_, a number *n*_tr_ of transit compartments *x*_tr_ with different levels of maturation, and a compartment *x*_ma_ with mature, circulating WBCs. Cells mature with a maturation rate *G*. Mature cells *x*_ma_ die by apoptosis with a death rate of *k*_ma_. The pharmacodynamic effect of Ara-C is described as a log-linear function *E* targeting the proliferating cells in the bone marrow. It depends on the concentration *x*_1_ of Ara-C in an assumed central compartment including the circulating blood. The proliferation rate *F* of *x*_pr_ models the replication speed of proliferating progenitor cells. Modelling assumptions were incorporated by choosing different functions *F* and *G* (compare Table 1). The estimated model parameters used for personalisation were *B*, slope, *k*_tr_, *γ*, and initial conditions.

Fig 1 illustrates the basic assumptions from which we derived twelve model variations of the original Friberg model which we denote by M1–M12 from now on. They differ concerning the number of transition compartments (M1–M3), initial conditions for the differential equations (M3–M5), and model assumptions for the possible effects of Ara-C on proliferation and maturation rates (M5–M12). In this designation, the original Friberg model is denoted by M2. The ordering of the first three models was chosen with respect to the decending number of transition compartments. Quartino *et al*. [30] proposed a model with six instead of three transition compartments and is thus denoted as M1. After intermediate evaluations of accuracies we concentrated on the most promising choice of scaling, transition compartments, and initial conditions, and included different modelling assumptions in the models M6–M12 which are alternatives to M5, our reference myelosuppression model extended to Ara-C. Most models refer to previous approaches in the literature and are included for a comprehensive comparison and evaluation of our new hypotheses.

#### M1–M5: The basic PK/PD model, number of compartments and initial conditions

In 2002 Friberg *et al*. published a PK/PD model describing myelosuppression induced by different chemotherapeutic agents (docetaxel, paclitaxel, and etoposide) [12]. The well studied model showed a good trade-off between capturing the important aspects of the dynamics, containing a moderate number of identifiable model parameters, and being applicable for different cytostatic drugs. It has become the gold-standard model in pharmacometrics [16] with different PK and population-based modifications to topotecan [31], to daunorubicin [32], to a combination therapy of Ara-C (low-dose), etoposide and daunorubicin in the induction treatment for AML [20], to a physiologically based PK model for the induction therapy of AML patients with daunorubicin and Ara-C (low-dose) [33], to a combination therapy of carboplatin, etoposide and thiotepa [34], to paclitaxel [22], to an individual-based approach [35], and to drug specific optimisations [21].

WBCs derive from differentiated, matured haematopoietic stem cells that have passed through several intermediate stages during maturation. The chain of maturation is reflected in the mathematical model as a clustering of cells in several consecutive compartments with identical properties. Each compartment is described as a differential state. The 4 + *n*_tr_ differential states of our mathematical models M1–M12 are the amounts *x*_1_ and *x*_2_ of Ara-C in two PK compartments, respectively, the amounts *x*_pr_ of proliferating cells, $x_{\text{tr},1},\ldots ,x_{\text{tr},n_{\text{tr}}}$ of differentiating cells in *n*_tr_ transient compartments, and *x*_ma_ of mature, circulating WBCs.

The differential equations that correspond to Fig 1 are

$${\dot{x}}_{1}\left(t\right)=-\left(k_{10}+k_{12}\right)\;x_{1}\left(t\right)+k_{21}\;x_{2}\left(t\right)+\frac{u(t)\;\text{BSA}}{\text{duration}}$$ (1)

$${\dot{x}}_{2}\left(t\right)=k_{12}\;x_{1}\left(t\right)-k_{21}\;x_{2}\left(t\right)$$ (2)

$${\dot{x}}_{\text{pr}}\left(t\right)=-G\left(k_{\text{tr}},x_{1}\right)\;x_{\text{pr}}\left(t\right)+F\left(x,k_{\text{tr}},\gamma ,B,\text{slope}\right)\;x_{\text{pr}}\left(t\right)$$ (3)

$${\dot{x}}_{\text{tr},1}\left(t\right)=G\left(k_{\text{tr}},x_{1}\right)\;x_{\text{pr}}\left(t\right)-G\left(k_{\text{tr}},x_{1}\right)\;x_{\text{tr},1}\left(t\right)$$ (4)

$$\begin{array}{cc} {\dot{x}}_{\text{tr},2}\left(t\right) & =G\left(k_{\text{tr}},x_{1}\right)\;\left(x_{\text{tr},1}\left(t\right)-x_{\text{tr},2}\left(t\right)\right)\\ & \ldots \end{array}$$ (5)

$${\dot{x}}_{\text{tr},n_{\text{tr}}}\left(t\right)=G\left(k_{\text{tr}},x_{1}\right)\;\left(x_{\text{tr},n_{\text{tr}}-1}\left(t\right)-x_{\text{tr},n_{\text{tr}}}\left(t\right)\right)$$ (6)

$${\dot{x}}_{\text{ma}}\left(t\right)=G\left(k_{\text{tr}},x_{1}\right)\;x_{\text{tr},n_{\text{tr}}}\left(t\right)-k_{\text{ma}}x_{\text{ma}}\left(t\right)$$ (7)

with constants *k*_10_, *k*_12_, *k*_21_, BSA, duration, *c*_*V*_, *k*_ma_, Ara-C administration u(t) and parameters *k*_tr_, *γ*, *B* and slope specified in the following model description and listed in S3 Table. Functions *F* and *G* are chosen differently in the models M1–M12, compare Table 1.

**Table 1:** Overview of all investigated mathematical models M1–M12.

Model	n_tr_	Initial condition	Proliferation rate F(x,k_tr_, *γ*, B, slope)	G(k_tr_,x_1_)	Parameters
**M1**	6	I1	(1 − *E*)*k*_tr_(*B*/*x*_ma_)^*γ*^	*k*_tr_	
Myelosuppression model with *n*_tr_ = 6 transition compartments, proposed in [19].					*B*, *k*_tr_, *γ*, slope
**M2**	3	I1	(1 − *E*)*k*_tr_(*B*/*x*_ma_)^*γ*^	*k*_tr_	
Original Friberg model [12] with *n*_tr_ = 3 transition compartments.					*B*, *k*_tr_, *γ*, slope
**M3**	1	I1	(1 − *E*)*k*_tr_(*B*/*x*_ma_)^*γ*^	*k*_tr_	
As M1, with *n*_tr_ = 1 transition compartments.					*B*, *k*_tr_, *γ*, slope
**M4**	1	I2	(1 − *E*)*k*_tr_(*B*/*x*_ma_)^*γ*^	*k*_tr_	*B*_0_
As M3, but with initial condition approach I2 resulting in 1 additional parameter.					*B*, *k*_tr_, *γ*, slope
**M5**	1	I3	(1 − *E*)*k*_tr_(*B*/*x*_ma_)^*γ*^	*k*_tr_	*x*_pr_(*t*_0_), *x*_tr_(*t*_0_), *B*_0_
As M3, but with initial condition approach I2 resulting in 3 additional parameters.					*B*, *k*_tr_, *γ*, slope
**M6**	1	I3	*k*_tr_(*B*/*x*_ma_)^*γ*^ − *E*	*k*_tr_	*x*_pr_(*t*_0_), *x*_tr_(*t*_0_), *B*_0_
As M5, but assuming a direct killing effect of Ara-C on the proliferating cells.					*B*, *k*_tr_, *γ*, slope
**M7**	1	I3	(1 − *E*)*k*_tr_/*S*(*x*_1_)(*B*/*x*_ma_)^*γ*^	*k*_tr_/*S*(*x*_1_)	*x*_pr_(*t*_0_), *x*_tr_(*t*_0_), *B*_0_
As M5, but replacing *k*_tr_ by *k*_tr_/*S*(*x*_1_) throughout.					*B*, *k*_tr_, *γ*, slope
**M8**	1	I3	(1 − *E*)*k*_tr_/*S*(*x*_1_)(*B*/*x*_ma_)^*γ*^	*k*_tr_	*x*_pr_(*t*_0_), *x*_tr_(*t*_0_), *B*_0_
As M5, but replacing *F* by *F*/*S*(*x*_1_).					*B*, *k*_tr_, *γ*, slope
**M9**	1	I3	(1 − *E*)*k*_tr_/*S*(*x*_1_)(*B*/*x*_ma_)^*γS*(*x*_1_)^	*k*_tr_	*x*_pr_(*t*_0_), *x*_tr_(*t*_0_), *B*_0_
As M8, but also multiplying *γ* with *S*(*x*_1_).					*B*, *k*_tr_, *γ*, slope
**M10**	1	I3	(1 − *E*)*k*_tr_(*B*/*x*_ma_)^*γS*(*x*_1_)^	*k*_tr_	*x*_pr_(*t*_0_), *x*_tr_(*t*_0_), *B*_0_
As M5, but multiplying *γ* with *S*(*x*_1_), possibly via macrophage activation.					*B*, *k*_tr_, *γ*, slope
**M11**	1	I3	(1 − *E*)*k*_tr_(*B*_bm_/(0.01 * *x*_pr_+ 0.99 * *x*_tr_))^*γ*^	*k*_tr_	*x*_pr_(*t*_0_), *x*_tr_(*t*_0_), *B*_0_
As M5, but feedback depends on bone marrow precursor WBC instead of WBC.					*B*, *k*_tr_, *γ*, slope
**M12**	1	I3	(1 − *E*)*k*_tr_(*B*_bm_/(0.01 * *x*_pr_+ 0.99 * *x*_tr_))^*γS*(*x*_1_)^	*k*_tr_	*x*_pr_(*t*_0_), *x*_tr_(*t*_0_), *B*_0_
Combining both modelling assumptions of M10 and M11.					*B*, *k*_tr_, *γ*, slope

For each mathematical model the number of transition compartments *n*_tr_, the initial condition strategy, and the two functions *F* for proliferation rate and *G* for maturation rate are specified, compare section *Methods* and Fig 1, respectively. The models M1–M5 have been used mainly to determine the best number of transition compartments and initial condition strategy, which have been kept fixed from M5 onward. Different modelling assumptions are incorporated via different functions *F* and *G* in the models M5–M12. An important role has the function *S*(*x*_1_) ≔ 1+ ln(1+ *c*_*V*_ *x*_1_), compare section *Methods*. Most important for this paper are the reference model M5 and the extended models M10 and M12 as the most promising PK/PD models in the context of Ara-C.

For fixed transition rate *k*_tr_, the number of compartments can be used to model the delay between the proliferating and circulating cells (mean maturation time [36]). As there is no common consensus on the precise number of differentiation stages [7, 37] we compared *n*_tr_ = 6, as proposed by Nock [34], *n*_tr_ = 3, as proposed by Friberg *et al*. [12] and *n*_tr_ = 1, proposed by us in which we comprise the whole maturation process into one transition compartment for models M5-M12. The fusion of the differentiation steps into one compartment is justified by the mean maturation time (MMT) from proliferating stem cells to circulating mature WBCs, which we compared with published values and presented in the section *Results*. Cells mature with a maturation rate constant *G* = *k*_tr_ summarising the fraction of cells performing self-renewal and differentiation into one parameter. This is a simplified assumption made by Friberg *et al*. guaranteeing homeostasis [17] and identifiability of the estimated parameters. During the modelling process we analysed a model considering separate parameters for the fraction of self-renewal and for differentiation in each compartment. The model has a similarly high accuracy but more challenging identifiability properties. Our findings are summarised in S5 Table.

Mature cells *x*_ma_ die by apoptosis with a death rate constant *k*_ma_. As Monte Carlo simulations were not very sensitive, we fixed *k*_ma_ to a constant value as previously proposed [19]. The time dependent dosage of Ara-C is denoted by *u*(*t*) and is specified by the individual treatment plan and the body surface area (BSA).

Each compartment represents the number of cells per liter. Liter is referred to the peripheral blood, so that the WBC counts from the last compartment coincide with the measured WBC counts from the clinical data. The model also determines the cell numbers of the upstream compartments. These numbers are provided per liter (peripheral blood) as well. If we are interested in the correct number of cells per liter in one of the compartments located in the bone marrow, we need a conversion factor from blood volume to bone marrow volume. We will not discuss this issue further and refer to [38]. Therein, the authors state that the functional haematopoietic marrow volume of about 1.75 L can increase sixfold depending on infection or haemorrhage.

We used a standard two-compartment PK model of high-dose Ara-C, which is administered in the consolidation phase, with zero-order input and linear elimination based on published drug concentration-time data [39]. The elimination rate constant *k*_10_, the transfer rate constants *k*_12_, *k*_21_ and the distribution volume of the central compartment *V*_*C*_ were estimated in a previous step and defined as constants for all further computations. A detailed discussion of the PK model is presented in the next section.

The PD effect, e.g. the negative effect of Ara-C on the proliferating cells, linking the PK model, especially the Ara-C concentration, to the myelosuppression model was modelled by a log-linear function *E* = slope ln(1+ *c*_*V*_ *x*_1_), using the parameter slope for patient-specific calibration and chemotherapeutical effects and the constant *c*_*V*_ for unit consistency (see S3 Table). We also implemented a linear PD function with discouraging results. Additionally, we tested a (sigmoid) *E*_*max*_ model without achieving better model accuracies. The three different PD functions are the commonly used mechanistic models describing pharmacodynamic effects in PK/PD modelling [40].

The function *F*(*x*, *k*_tr_, *γ*, *B*, slope) is a general description of the proliferation rate of *x*_pr_ and incorporates the PD effect *E* on the proliferating cells, as discussed in Minami *et al*. [41], Derendorf *et al*. [42] and applied, e.g. in Hing *et al*. [43]. The basic structure of the function *F* derived in [12], is (1 − *E*)*k*_tr_(*B*/*x*_ma_)^*γ*^ in which the mature cells influence the proliferation rate *k*_tr_ of *x*_pr_ with a feedback term (*B*/*x*_ma_)^*γ*^ that leads to higher rates if the number of circulating cells *x*_ma_ is below the baseline WBC count *B*, and vice versa. It is motivated from studies showing that the proliferation rate can be affected by endogenous growth factors and cytokines [44] and that circulating neutrophil counts and the growth factor G-CSF levels are inversely related [45]. Including this term allows a temporary overshoot of WBC compared with the baseline value *B*. The proliferation exponent *γ* indicates the strength or speed of this feedback. The estimation parameters were *B*, slope, *k*_tr_, and *γ* plus a varying number of additional parameters depending on the initial condition approach, see Table 1. The different initial condition approaches are introduced in the next sections. The WBCs completely recover after cytotoxic therapy (see Fig 2a in [46]) and each cycle is scheduled such that the WBCs should be in homeostasis before treatment start. However, our clinical data indicate that not for all patients the WBCs are yet completely recovered from myelosuppression or they already recovered and overshoot their steady state value before the start of the next treatment cycle due to an overproduction of WBCs. This *carry over effect* was already mentioned by Kloft *et al*. [31] but was not considered by them. Thus, we implemented three different strategies to treat initial conditions: I1 assumes a steady state, I2 assumes a steady state only for the proliferating and transient compartments, and I3 penalises deviations from the steady state. I2 and I3 are considered as alternative initial conditions as our clinical data indicate that the steady state assumption after induction phase and between the consolidation cycles may be violated.

We used I1 and *n*_tr_ = 6 for M1, I1 and *n*_tr_ = 3 for M2, I1 and *n*_tr_ = 1 for M3, I2 and *n*_tr_ = 1 for M4, and I3 and *n*_tr_ = 1 for M5–M12. Model constants, patient-specific constants and units of model parameters are summarised in S3 Table.

Apart from different PK models which were linked to the myelosuppression model, modifications of the structural model were also proposed [22, 23]. Both models have a more detailed description of the stem cell compartment. The model from Henrich *et al*. covers a consecutive decrease of the leukocyte’s nadir in the treatment cycles achieved by a prior additional compartment mimicking the slow replication of pluripotent stem cells in the bone marrow. Mangas-Sanjuan *et al*. models a cell-cycle occurring in the bone marrow compartment covering quiescent cells which do not enter the proliferation process and are not sensitive to the pharmacodynamic effect of the treatment.

#### Development of PK Model

**Fig 2:**
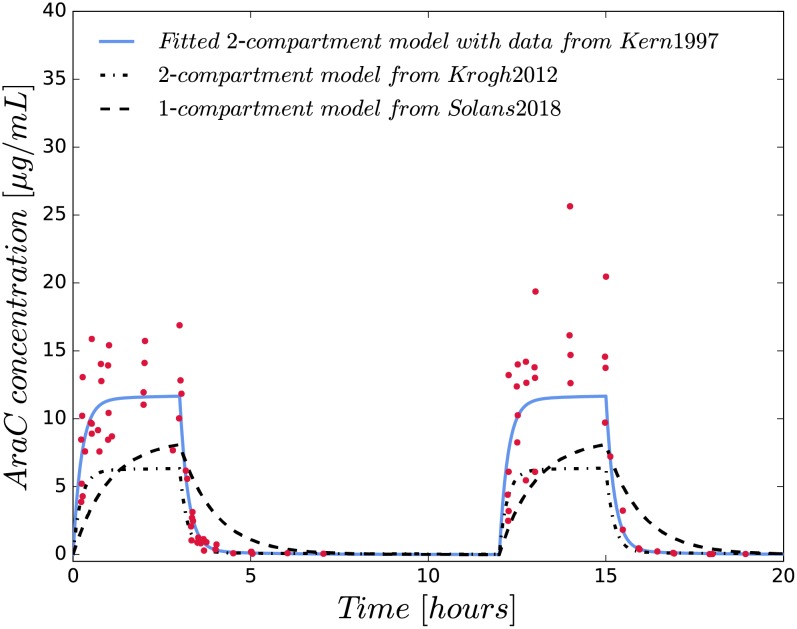
Simulations of different pharmacokinetic models and Ara-C concentration measurements from Kern *et al*. [39].

We developed a two-compartment PK model for intraveneous high-dose Ara-C infusions and compared it with two previously published PK models [20, 47]. 86 Ara-C concentration measurements (*μg*/*mL* = *mg*/*L*) from 11 patients were collected from Fig 2 of Kern *et al*. [39] and presented in Fig 2.

As Fig 2 in [39] was our only source of data, no inter–individual variability analysis could be performed. The patients received high-dose (3 *g*/*m*^2^) Ara-C infusions over 3 hours every 12 hours on days 1, 2, 8 and 9. The measurements we are using were collected at day 1 and 8 and we assume BSA = 1.78 *m*^2^. The resulting model with unknown parameters *k*_10_, *k*_12_, *k*_21_ and *V*_*c*_ denoting the elimination rate, distribution rates and the volume of the central compartment, is formulated as

$${\dot{x}}_{1}\left(t\right)=-\left(k_{10}+k_{12}\right)\;x_{1}\left(t\right)+k_{21}\;x_{2}\left(t\right)+\frac{u(t)\text{BSA}}{3}$$ (8)

$${\dot{x}}_{2}\left(t\right)=k_{12}\;x_{1}\left(t\right)-k_{21}\;x_{2}\left(t\right).$$ (9)

We estimated the unknown parameters using a naive pooling approach with exponential error model $\eta_{ij}=\frac{x_{1}\left(t_{ij}\right)}{V_{c}}\;e^{\varepsilon_{ij}}$ where $\varepsilon_{ij}∼N\left(0,1\right)$. The naive pooling approach was used as the collected Ara-C measurements could not be assigned to the corresponding patients. The error model is transformed to $log\left(\eta_{ij}\right)=log\left(\frac{x_{1}\left(t_{ij}\right)}{V_{c}}\right)+\varepsilon_{ij}$ and the following parameter estimation problem

$$\begin{array}{ccc} \underset{k_{10},k_{12},k_{21},V_{c},x}{\text{min}} & & \frac{1}{2}\sum_{i=1}^{m}\sum_{j=1}^{n_{i}}{\left(\;log\left(\eta_{ij}\right)-log\left(x_{1}\left(t_{ij}\right)/V_{c}\right)\;\right)}^{2} \end{array}$$ (10)

s.t.x˙(t)0=f(x(t),u(t),k10,k12,k21,V1),x(t0)=(0.,0.)T (11)

is solved with a Gauss-Newton algorithm implemented in CasADi [48] with single shooting (CVODES). The estimated parameters and their relative standard deviations are presented in Table 2 together with the parameter values from Solans *et al*. [47] and Krogh *et al*. [20].

**Table 2:** Comparison of our derived PK model with a published one- and two-compartment model.

	Solans2018 [47]	Krogh2012 [20]	Ours
*CL*[*L*/*h*]	208.73	272.0	154.225
*V*_*p*_ [*L*]	-	75.4	7.7825
*Q* [*L*/*h*]	-	13.7	4.1761
*V*_*c*_ [*L*]	209.25	62.8	37.6571(21.30%)
*k*_10_[1/*h*]	1.0	4.3	4.0955(15.09%)
*k*_12_[1/*h*]	-	0.2	0.1109(67.64%)
*k*_21_[1/*h*]	-	0.2	0.5366(69.50%)

Final parameter estimates and relative standard errors (in brackets) are shown. Comparing our model with a published two-compartment model for low-dose Ara-C [20], we estimated a smaller central volume leading to a reduced clearance activity derived from an almost equivalently estimated elimination rate constant value. The distribution rate constants differ by a factor of 2 to 2.5 and the peripheral volume by a factor of almost 10. The parameter values and the visual assessment of the one-compartment model [47] in S8 Fig indicate, that the one- and two-compartment models describe Ara-C concentrations with qualitatively different dynamics.

The two-compartment PK model representing a central and peripheral compartment, see Fig 1, adequately described the concentration-time data and coincides with the derived values for clearance and the elimination rate constant *k*_10_ from Table 6 in [39]. We used our derived two-compartment model with the given estimated parameter values in all calculations. We did not use the PK models from Krogh *et al*. and Solans *et al*., because they were fitted to low-dose treatment schedules. Although Ara-C is reported to have a linear pharmacokinetics [39], simulations with these PK models did not coincide well with the concentration measurements from high-dose treatment schedules published in [39] as shown in Fig 2.

During the model development process we also tested a K-PD approach [49] which resulted in non-identifiability of the elimination rate constant of the virtual compartment during the individual parameter estimations.

As stated above the PK model was fixed in the modelling process. In one of the simulation studies we analysed the effect of the PK variability on the different modelling hypotheses. We present and discuss the results in S8 Fig.

#### M6: Modelling a direct killing effect of Ara-C on the proliferating cells

In the model M6, we chose the proliferation rate as discussed in previous works [21, 33, 42] as *F* = *k*_tr_(*B*/*x*_ma_)^*γ*^ − *E*. The main difference to all other models is that the PD effect *E* is directly multiplied with *x*_pr_ and not with *k*_tr_(*B*/*x*_ma_)^*γ*^*x*_pr_. Multiplying with *x*_pr_ can be seen as a direct (killing) impact of Ara-C on the amount of proliferating cells, whereas the more plausible mechanism-based rationale is the induced reduction of the proliferation rate constant *k*_tr_ used in all models except in M6.

#### M7–M12: Extending the effects of Ara-C

The root mean squared error (RMSE) values indicate that model M5 with one transition compartment and initial condition approach I3 (described in the next section) provides the highest accuracy after model personalisation compared to M1–M4.

The indirect effect of Ara-C with an impaired proliferation (M5) is more plausible than a direct killing effect (M6), because Ara-CTP is incorporated into DNA and RNA and impairs cell replication [26]. Therefore, M5 became the reference model for all further analysis. We extended the proliferation rate *F*(⋅) and/or the transition rate *G*(⋅) in M5 to capture potential secondary effects of Ara-C. To understand the implications of the extensions, we observe that the proliferation rate *F* = (1 − *E*) *k*_tr_(*B*/*x*_ma_)^*γ*^ is negative when 1 < *E*. This is the case for

$$c_{V}\;x_{1}>e^{{\text{slope}}^{-1}}-1.$$ (12)

This corresponds to more proliferating cells being in the process of apoptosis than being in the process of cell division. It is important that the feedback term (*B*/*x*_ma_)^*γ*^ increases the absolute value of *F* for *B* > *x*_ma_, and decreases it for *B* < *x*_ma_. Therefore, an analysis of *F* always has to consider all four cases related to the signs of 1 − *E* and of *B* − *x*_ma_. Inspired by the log-linear behaviour of the PD effect *E*, we chose

$$S\left(x_{1}\right)≔1+\text{ln}(1+c_{V}\;x_{1}).$$

This monotonously increasing function is applied to different expressions in M5.

In M7 we replaced the transition rate *k*_tr_ by *k*_tr_/*S*(*x*_1_) throughout M5. This results in an Ara-C induced reduction of the transition rate.

In M8 we replaced the complete feedback function *F* in M5 by *F*/*S*(*x*_1_). This models an Ara-C induced decreased auto-feedback of the proliferating cells. For high values of *x*_1_, i.e. when (12) holds, this results in a decreased killing of proliferative cells. For values *x*_1_ > 0 below that boundary, we get a decreased positive proliferation rate.

In M9 we replaced both the complete feedback function *F* by *F*/*S*(*x*_1_) and the proliferation exponent *γ* by *γ S*(*x*_1_). Again, depending on *x*_1_ either the killing or the proliferation rate of *x*_pr_ are decreased by *F*/*S*(*x*_1_). In addition, the impact depends on whether the WBC count is below or above the baseline: for *x*_ma_ < *B* we have an increased killing/proliferation rate (*B*/*x*_ma_)^*γ S*(*x*_1_)^ > (*B*/*x*_ma_)^*γ*^ and vice versa.

In M10 we replaced the proliferation exponent *γ* in M5 by *γS*(*x*_1_). This is motivated by the observation that the feedback term with exponent *γ* is related to the endogenous G-CSF [12]. In contrast to M9 the function *F* itself is not scaled. Like in M9, the *γS*(*x*_1_) scaling results in an increase of killing/proliferation rates for WBC counts below the baseline, and a decrease else.

In M11 we replaced the quotient *B*/*x*_ma_ by a comparison between cells in the bone marrow and their baseline value. Based on the statement in [7] and the references therein, we assumed that about 1% of the WBC precursor cells in the bone marrow are in the proliferating compartment *x*_pr_, and 99% in the transition compartment *x*_tr_. In M12 we combined the extensions from M10 and M11.

The parameter vector contains also initial values, which we discuss next.

### Initial conditions of the differential states

The initial values of the PK were chosen to be zero, *x*_1_(*t*_0_) = *x*_2_(*t*_0_) = 0, due to the fact that the considered time horizons start before administration of chemotherapy. Further, it is known that previous Ara-C treatments have no impact on the pharmacokinetics of subsequent treatments [39] which is supported by simulation studies showing that the values of *x*_1_(*t*) and *x*_2_(*t*) are below 10^−6^ after 16.35 days of the 1 Ara-C infusion. The remaining initial conditions were chosen using one of the following three strategies.

#### Initial condition approach I1

The WBC count *x*_ma_(*t*_0_) was set to the long term WBC baseline (steady state) count *B*. With this particular choice all feedback terms simplify to

$$k_{\text{tr}}{\left(B/x_{\text{ma}}\left(t_{0}\right)\right)}^{\gamma }=k_{\text{tr}}.$$

Assuming *x*_1_ = 0, also *E* = 0, *S*(*x*_1_) = 1, and hence *F* = *G* = *k*_tr_ for all models at time *t*_0_, which simplifies the analysis. As initial conditions for the cell counts in the bone marrow we chose the bone marrow baseline cell count $B_{\text{bm}}≔\frac{B\;k_{\text{ma}}}{k_{\text{tr}}}$,

$$x_{\text{pr}}\left(t_{0}\right)=B_{\text{bm}}$$ (13)

$$x_{\text{tr},1}\left(t_{0}\right)=\ldots =x_{\text{tr},n_{\text{tr}}}\left(t_{0}\right)=B_{\text{bm}},$$ (14)

which guarantees that inserting (13) into (1) leads to a right hand side of zero.

The advantage of this approach is the identifiability of the estimation problem, as no additional degrees of freedom in the estimation problem need to be introduced for the initial conditions. However, simulations showed that both the assumption of *x*_ma_(*t*_0_) = *B* and the steady state assumption were typically violated at the beginning of a new consolidation cycle.

#### Initial condition approach I2

One additional parameter *B*_0_ was introduced and estimated, as suggested by Nock [34]. It was used for the initialisation as

$$x_{\text{ma}}\left(t_{0}\right)=B_{0},$$ (15)

together with (14). The time derivative (1) at time *t*_0_ is given by

$${\dot{x}}_{\text{pr}}\left(t_{0}\right)=(k_{\text{tr}}(\frac{B}{B_{0}}{)}^{\gamma }-G\left(k_{\text{tr}},x_{1}\right))\;B_{\text{bm}},$$ (16)

$$\begin{array}{cc} {\dot{x}}_{\text{tr},1}\left(t_{0}\right)=\ldots ={\dot{x}}_{\text{tr},n_{\text{tr}}}\left(t_{0}\right) & =0 \end{array}$$ (17)

$${\dot{x}}_{\text{ma}}\left(t_{0}\right)=G\left(k_{\text{tr}},x_{1}\right)\;B\;k_{\text{ma}}/k_{\text{tr}}-k_{\text{ma}}B_{0},$$ (18)

which is not zero for *B*_0_ ≠ *B*. The advantage of this approach is that also increasing or decreasing WBC counts at *t*_0_ can be captured, depending on the sign of (18).

#### Initial condition approach I3

The initial conditions $x_{\text{pr}}\left(t_{0}\right),x_{\text{tr},1}\left(t_{0}\right),\ldots ,x_{\text{tr},n_{\text{tr}}}\left(t_{0}\right)$, and, as in I2, also *x*_ma_(*t*_0_) = *B*_0_ were introduced as additional estimation parameters. As this leads to unidentifiability of the estimation problem, a term penalising deviations from (13),

$$\alpha (x_{\text{pr}}\left(t_{0}\right)-B_{\text{bm}}{)}^{2}+\alpha \sum_{i=1}^{n_{\text{tr}}}(x_{\text{tr},i}\left(t_{0}\right)-B_{\text{bm}}{)}^{2}$$

with *α* = 1/2500 was added to the objective function of the least squares estimation problem. The regularisation parameter *α* was chosen with respect to the tradeoff between identifiability of *x*_pr_(*t*_0_) and *x*_tr,*i*_(*t*_0_) and the violation degree of the steady state assumption. Larger values of *α* resulted in similar parameter estimates compared to the initial condition approach I1, as more attention was drawn to the penalising terms guaranteeing a solution close to the steady state assumption after parameter estimation. Smaller values of *α* weakened the steady state assumption but also increased the uncertainty of parameter estimates for *x*_pr_(*t*_0_) and *x*_tr,*i*_(*t*_0_) resulting in large standard deviations. A good tradeoff was achieved with *α* = 1/2500. For more information about the regularisation approach see [50] and the references therein. Note that *B*−*B*_0_ was not penalised. This approach is the most flexible with respect to the possibly transient initial dynamics resulting, e.g. from previous treatments.

### Clinical data (high density WBC counts) & personalisation

AML patients who had received induction therapy (commonly defined as anthracycline- and Ara-C-based *7+3 regimen* [2]) resulting in complete remission and who did not receive granulocyte-colony stimulating factors (G-CSF) during the post-remission consolidation therapy were eligible for data analysis. We focused on patients who did not receive growth factor support, as such effects were not yet accounted for in our mathematical models. Almost daily WBC counts from 42 consolidation Ara-C cycles (CCs) of 23 AML patients (median 62 years, 14 male, mostly *de novo* AML (19/23), mostly AML FAB-M2 (9/19), mostly intermediate cytogenetic risk (12/20)) from 2008 to 2015 were analysed from clinical charts provided by the Department of Hematology and Oncology, Magdeburg University Hospital, Magdeburg, Germany. The data were retrospectively collected and pseudonymised from records of the clinical routine, and interventions were not performed for this work. For this reason no patients’ agreements were required. This study was approved by the Ethics Committee of the Medical Faculty of the University of Magdeburg (Optimal control of clinically relevant cancer chemotherapy schedules in patients with acute leukaemia—with special emphasis on neutropenia, MARTINA; approval number 124/15). All clinical procedures were performed in accordance with the general ethical principles outlined in the Declaration of Helsinki. The CCs were partitioned in one, two, and three consecutive CCs from nine, nine, and five patients, respectively. Four different schedules D135, d135, D123, or D12, in which the numbers correspond to treatment days 1, 2, 3, and 5, respectively, d to intermediate-dose Ara-C (i.e. 1 *g*/*m*^2^ per BSA twice a day over three hours) and D to high-dose Ara-C (i.e. 3 *g*/*m*^2^ twice a day), were administered 23, 15, two, and two times. Patient *P*_D123_ (62 years, male) received two cycles of D123. Patient *P*_D12_ (64 years, female) received two cycles of D12. The 21 other patients received 1-3 D135 cycles (median 57 years, 8 male, 4 female) or d135 cycles (median 68 years, 5 male, 4 female).

The cycle- and patientwise longitudinal WBC count measurements are published as NONMEM compatible comma separated values files in the supporting information (S1 and S2 Files). Both datasets contain the columns ID TIME DV CMT AMT RATE DUR MDV and EVID. ID serves as an identifier for the appropriate cycle, respectively patient. In the cyclewise dataset each of the 42 CCs has its individual ID meaning that each cycle is treated independently although several cycles belong to the same patient. In the patientwise dataset all cycles belonging to the same patient are assigned to one ID. TIME [day] either specifies the measurement times of WBC counts or the starting times of the Ara-C administrations. DV [G/L] is the dependent variable, in our case individual WBC count measurements. The column CMT specifies the compartment in which a dosing or observation event occurs. AMT [mg], RATE and DUR [day] define the Ara-C schedules. As every administration is an infusion lasting 3 hours, the entries of DUR and RATE are 0.125 [day] and -2. AMT defines the BSA-adjusted amount [mg] of Ara-C. The column MDV allows the user to inform NONMEM whether or not the value in the DV field is missing, but in our case the datasets do not contain missing measurements. The column EVID explicitly declares to NONMEM the type of the current record. EVID = 0 defines the record as an observation event and EVID = 1 defines the record as a dose event. For more information about NONMEM datasets we refer the interested readers to [51].

We used all 42 CCs to personalise our mathematical models M1–M12 performing point estimations (individual approach). The point estimates were used to analyse the different modelling assumptions. Additionally we personalised the most relevant models M3, M10 (with I1), the model from Henrich *et al*. [22], the model from Mangas-Sanjuan *et al*. [23] and the model from Stiehl *et al*. [8] applying nonlinear mixed-effects modelling (population approach). The population approach is used on the one hand to qualitatively confirm our proposed model variation based on the set of population parameters. On the other hand we wanted to compare our set of population parameter values with recently published models and give a reason why the Friberg model serves as our basic model and not recently published models which are similar to the Friberg model, but with several extensions. A comment on the use of initial value approach I1 instead of I3 for the population approach is given in the S1 Appendix. Once the model parameters have particular values, the model is called personalised model (PM).

### Prediction & cross-validation

The PMs were then used to predict (simulate) and cross-validate WBC counts for the last CC of 14 patients for whom at least two consecutive CCs are available. Additionally, we calculated predicted *t*_rec_ values from our 42 PMs applying D123 and D135 schedules and compared the descriptive statistics with published average *t*_rec_ values from a subset of data (367 CCs of 208 AML patients, no G-CSF support) of the AMLSG 07-04 trial in which the schedules D123 and D135 after *7+3 regimen* were analysed [46]. The published AMLSG 07-04 [46] trial does not provide WBC counts to obtain new PMs, therefore we used the median of observed *t*_rec_ values for D123 and D135 Ara-C schedules. In the interest of a fair comparison (i.e., to avoid comparison with the value 0) we excluded five (d135:1 and D135:4) out of 42 PMs for which at least one out of the 42 predictions (M1–M12 with either D123 or D135) resulted in no WBC counts below the threshold value. This could occur as we personalised the models for a specific treatment plan, e.g. D135. Afterwards we applied a different treatment plan to the PMs, i.e D123, which may have resulted in a reduced cytotoxic effect. Not each out of the 42 predictions resulted in a nadir value below 1 *G*/*L*. Further, we predicted *t*_rec_ values for two Ara-C schedules in which a constant administration of Ara-C throughout days 1-5, with either 100 *mg*/*m*^2^ per day or 400 *mg*/*m*^2^ per day was given. These schedules, together with D135, have been clinically analysed for 1088 AML patients (median 52, 568 male) by Mayer *et al*. [3], and the superiority of D135 with respect to disease-free survival rates and remaining in continuous complete remission after four years has been shown but no *t*_*rec*_ values were reported. Finally, we analysed the effect of the inter-individual PK variability on the *t*_*rec*_ values derived by the models M3 and M10 (with I1). We applied schedules D123 and D135 with fixed population parameter values for *B*, slope, *k*_tr_, and *γ* and performed 500 simulations each with randomly chosen values from constructed inter-individual variability (IIV) for the PK parameters clearance *CL* and central volume *V*_*C*_.

All experiments were performed to analyse the 12 proposed models with respect to WBC count and *t*_rec_ predictability.

### Schedule timing

After verifying the predictability performance of the PMs, we performed a simulation study in which we demonstrated a further possible application of the PMs in planning the start of consecutive CCs. We analysed the impact of the treatment timing on the individual nadir values. For each of the 14 patients, for whom at least two consecutive CCs were available, the nadir of the last CC was compared to 20 simulated nadirs. These nadirs resulted from simulations using the patient’s PMs (second row of Table 3) in which the timing of the last CC was varied daily with the maximal starting variation of 10 days earlier or later.

**Table 3:** Root mean squared error (RMSE) values for the models M1–M12.

RMSE	M1	M2	M3	M4	M5	M6	M7	M8	M9	M10	M11	M12
42 CCs	0.911	0.836	0.742	0.636	0.579	0.595	0.639	0.576	0.574	0.574	0.577	0.587
23 Pat	1.154	1.011	0.892	0.825	0.741	0.758	0.785	0.753	0.738	0.740	0.740	0.741
14 Pred	1.269	1.128	1.059	1.007	0.908	0.972	0.997	0.960	0.958	0.927	0.940	0.947
7 D135	1.108	0.912	0.834	0.778	0.750	0.753	0.781	0.750	0.767	0.765	0.731	0.768
5 d135	1.319	1.240	1.141	1.093	0.921	1.095	1.023	1.068	1.043	0.957	1.037	1.009
*P*_D123_	2.404	2.218	2.241	2.258	2.011	2.014	2.324	2.006	1.996	1.996	2.029	2.022
*P*_D12_	1.014	0.991	1.042	0.924	0.842	0.843	1.049	0.839	0.840	0.843	0.824	0.823

Measured and calculated WBC counts were compared. The estimations and predictions used personalised mathematical models (PMs) that were calculated based on the twelve different mathematical models M1–M12. The first row refers to a personalisation for all 42 consolidation cycles (CCs). The second row shows results for personalisations using all available cycles per patient (Pat). For predictions (Pred) all but one cycle were used for personalisation and the last cycle for cross-validation. Four more rows show the predictions sperated into the different schedules (D135, d135, D123 and D12). The RMSE values decrease from cycles to patients and from personalisation towards prediction, as expected. Comparing the mathematical models, the accuracy increases with a reduced number of compartments from M1 to M3. The initial condition strategies I2 in M4 and I3 in M5 decrease RMSEs further. M5–M12 all used *n*_tr_ = 1 and I3 and performed equally well, with the slight exception of M7. Note that in particular there is no significant difference between the established gold-standard model M5 and our newly proposed extended model M10.

## Results

### Accuracy of PMs with fixed Ara-C schedule

Table 3 shows statistics about the accuracies of the PMs describing the clinical data, for a pure estimation (using all available WBC counts to personalise the model) and for a cross-validation (using all but the last CC for personalisation).

**Fig 3:**
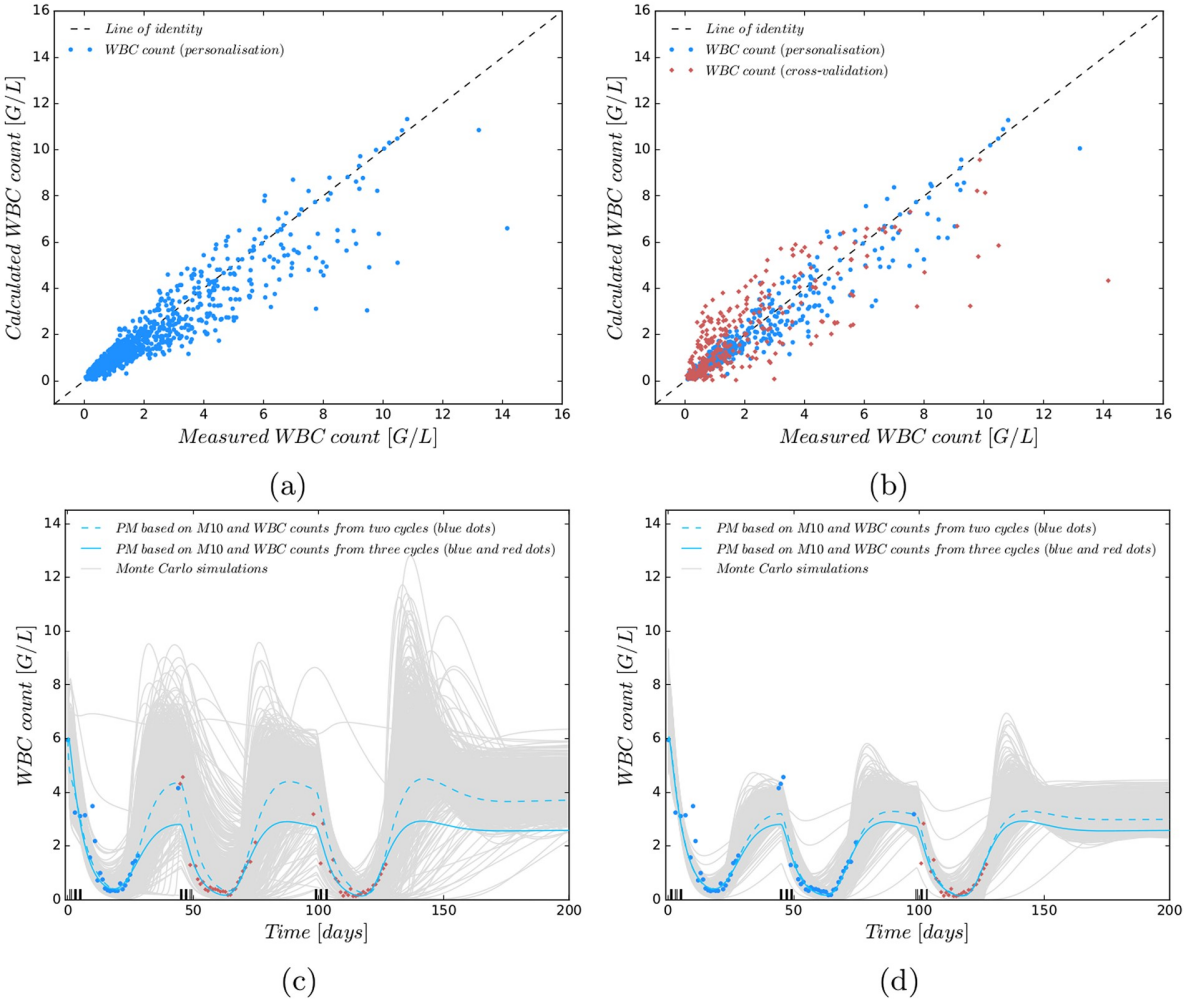
Visualisation of predictive accuracies of personalised mathematical models (PM). (**a**) Goodness-of-fit plot for M10. Shown are measured versus calculated white blood cell (WBC) counts. Models were personalised using complete data sets of one to three cycles from 23 patients. The measured counts around the nadir coincide well (RMSE = 0.740) with the calculated WBC counts. (**b**) As (a), but cross-validated: WBC counts from the last cycle of patients were not used for personalisation, but compared to predictions (RMSE = 0.927). The plot shows cross-validated WBC counts from the last cycle in red, others in blue. The plots are prototypical for M1–M12. (**c**) PMs based on M10 and either personalisation with WBC counts from one or from all three cycles. 1000 Monte Carlo simulations after personalisation with WBC counts from one cycle were used to indicate the propagated probability density function. (**d**) As (c), but using WBC counts from the first two cycles for personalisation. More measurements lead to higher prediction accuracy. The uncertainty tube tightens and the predicted trajectory gets closer to the solution that used all available WBC counts.

**Fig 4:**
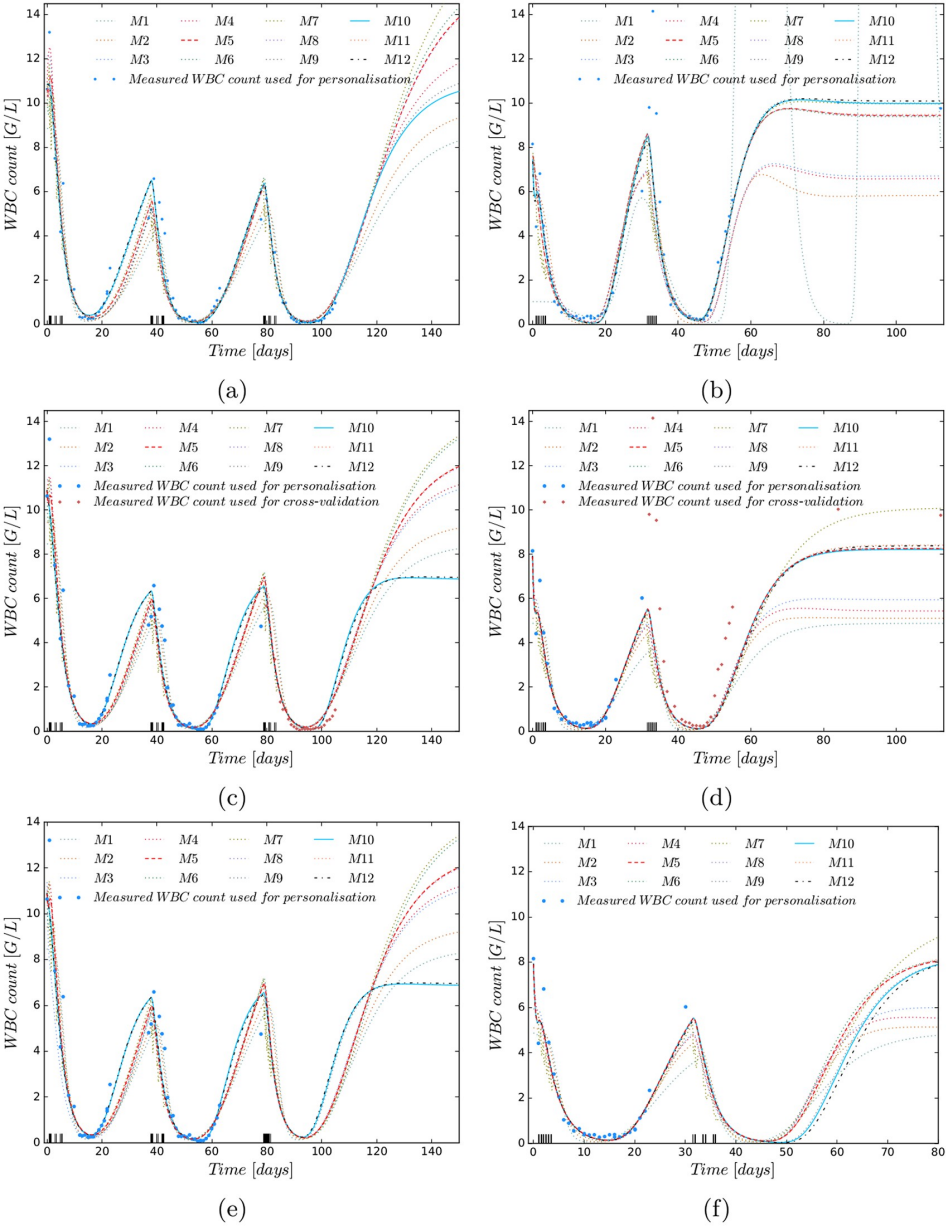
Comparison of personalised models (PMs) based on M1-M12 and white blood cell (WBC) data. Patient with three D135 cycles (left) and patient *P*_D123_ with two D123 cycles (right), as indicated on the x-axis. The PMs exemplify reproducability (first row), predictability (second row) and simulation of a different schedule in prediction than estimation (third row). (**a**) Reproducability: all 12 PMs based on M1–M12 are able to explain the measured WBC counts. (**b**) As in (a), all PMs explain the measured WBC counts well, particularly around the nadirs. (**c**) Cross-validated prediction: all PMs explain the WBC counts well, also in the predicted third cycle. (**d**) As in (c), here with a slightly too slow predicted recovery time in the second cycle for all models. **(e)** Varied Ara-C schedule: prediction of D123 in the third cycle for a PM based on two D135 cycles shows faster WBC recovery for M9, M10, and M12. **(f)** Prediction of D135 in the second cycle for a PM based on one D123 cycle shows slower WBC recovery times for M9, M10, and M12.

The accuracies depend strongly on the number of compartments and initial condition strategy (M1–M5), but do not differ much with respect to modelling assumptions of possible effects of Ara-C considered in M6–M12. These values were even better when the standard schedule D135 was applied in the estimated and predicted cycles. Regarding the root mean squared errors for M1-M5 the results implied that one transition compartment and initial approach I3 were the best choice for the structural model and hence served as a starting point to analyse different pharmacodynamic effects of Ara-C. As mentioned in the previous section, the number of transition compartments determines the MMT of the differentiating progenitor cells. Comparing the MMTs resulting from the population approach for M1-M3 we achieved a slight decrease from 154 h to 144 h to 128 h by using the corrected formula MMT = *n*/*k*_tr_[17] instead of the original formula MMT = (*n* + 1)/*k*_tr_ [12]. During the administration of cytostatic drugs it is known that the cells are encouraged to rapidly differentiate such that a MMT of 128 h is reasonable. Furthermore, the MMT value from one transition compartment is closest to a previously published corrected MMT value of 106.4 [17, 30]. Studies with healthy volunteers reported MMTs of 153.6 h and 165.6 h [17]. But these values are difficult to compare as chemotherapy can speed up proliferation and differentiation. With this knowledge and the accuracy values of Table 3 we decided to fix the number of transition compartments to one. The original MMT formula from [12] would have resulted in 180 h, 193 h and 256 h. By using M5 as the reference model and analysing different hypotheses of Ara-C’s PD effect in M6-M12, all models could describe the clinical data equally well. Goodness-of-fit plots in Fig 3a and 3b and S1 Fig visually support the good match between model predictions and measured WBC counts (respectively observed vs. calculated *t*_*rec*_ values) around the nadir and a wider spread of large WBC counts. To analyse the reliability of the PMs to predict the WBC dynamics in subsequent CCs, Fig 3c and 3d indicate the involved model uncertainty from parameter uncertainty by means of Monte Carlo simulations. The model uncertainty was derived from 1000 randomly chosen parameter sets sampled from the variance-covariance matrix resulting from the individual parameter estimation problem (10) (for more information see S1 Appendix). The information from one CC and no available prior knowledge leads to a high uncertainty. The uncertainty reduces when more WBC counts are present, and the prediction accuracy for consecutive CCs and myelosuppression increases. Examining the accuracy of the PMs for each patient separately, the WBC counts around the nadir are explained well by all models for fixed Ara-C schedules (either D135 or D123), as shown in Fig 4a–4d for two exemplary patients and in S2 and S3 Figs for the other 12 patients with at least two consecutive CCs.

Regarding the estimated parameter values, we only determine a slight change of the estimated fixed-effects parameter values for *B*, *k*_tr_ and slope, the inter-individual variability for all four parameters and the residual error between models M3 and M10 whereas the estimated fixed-effects parameter value for slope significantly decreases when a second PD term is introduced (see).

### Accuracy of PMs with altered Ara-C schedule

As we were interested to differentiate between the distinctive model hypothesis, we applied different chemotherapy schedules to the PMs and analysed their dynamical behaviour, especially the WBC recovery. Fig 4e and 4f show two cases where D135 was used for personalisation and D123 for prediction (and vice versa). Here, M9, M10, and M12 have a faster (slower) haematological recovery for D123 (D135). All three models assume that the proliferation speed *γ* depends on the Ara-C concentration. This modelling assumption is visualised in a different way for M5, M10 and M12 in S4, S5 and S6 Figs.

It is shown that the negative proliferation rate *F* of M10 and M12 compared to M5 has an altered dynamical behaviour during chemotherapy due to the increased *γ* value from the PD effect achieving a faster WBC recovery for D123 schedules. For this accelerated feedback relationship between WBCs and G-CSF biological interpretations are given in the next section.

In conclusion, the comparison of WBC recovery times between D123 and D135 treatments is a suitable criterium for model discrimination.

The next study was performed to compare the calculated *t*_*rec*_ values from M1-M12 with clinically collected values to figure out which of the models coincided with clinical findings. We used 444 PMs (using M1–M12 and clinical data from 37 cycles with schedules D135, d135, D123 and D12 from section *Clinical Data & Personalisation*) to predict the outcome of D135 and D123 schedules. The median values of the predicted *t*_rec_ were compared to the values from a subset of data (108 with D135 and 259 with D123 schedules) from the AMLSG 07-04 trial [46]. M9, M10, and M12 resulted in roughly 4 days faster *t*_rec_ for D123 compared to D135, similar to the clinical result from the literature and in contrast to the 1 day difference of M5 (compare Table 4). The individual results have been qualitatively confirmed by the predicted *t*_rec_ values from the population approach (see). The models from Henrich *et al*. and Mangas-Sanjuan *et al*. were not further considered, as both models simplified to the Friberg model after parameter estimation. For the model from Henrich *et al*., the estimated population parameter value *f*_*tr*_ was 0.96, supporting the visual assessment that the patients’ nadirs are not decreasing during the CCs. The estimated parameter values *k*_*cycle*_ = 0.0009 and *F*_*prol*_ = 0.941 of the model from Mangas-Sanjuan *et al*. yielded a non-existing stem cell cycle. A possible reason for the non-identifiability of the parameters might be the limited schedule variation. The authors state that a vast variation of schedules has to be available for parameter identification [23]. The model from Stiehl *et al*. provided the highest model accuracy with respect to the final objective function value, but exhibited disagreeing WBC recovery times and large relative standard errors (see S5 Table). Therefore we did not consider this model in our further studies. The simulation study analysing the effect of the PK variability on the resulting recovery times of schedules D123 and D135 for models M3 and M10 (with I1) revealed that model M10 was more sensitive to different high-dose Ara-C treatment schedules compared to model M3 despite the high inter-individual PK variability. This was verified in S8 Fig presenting boxplots of 500 simulated *t*_*rec*_ values for both models and schedules with constructed IIV on the PK.

**Table 4:** Double cross-validation with clinical data from two independent clinical trials.

		t_rec_				$t_{\text{rec}}^{D135-D123}$		t_leu_				nadir			
		**D123**		**D135**				**D123**		**D135**		**D123**		**D135**	
Clinical Data		19.3	±1.0	23.1	±2.9	–	–	11.5	±3.5	12.5	±4.7	0.3	±0.0	0.3	±0.2
*n*^*D*123^ = 2,*n*^*D*135^ = 23		(18.6	20.1)	(14.1	27.1)	–	–	(9.1	14.0)	(5.0	20.0)	(0.24	0.27)	(0.1	0.8)
Subdata (AMLSG 07-04)		18.0	–	22.0	–	–	–	–	–	–	–	–	–	–	–
*n*^*D*123^ = 259,*n*^*D*135^ = 108		(–	–)	(–	–)	–	–	–	–	–	–	–	–	–	–
**Model**	**Group**	**D123**		**D135**				**D123**		**D135**		**D123**		**D135**	
M1	37 PMs	21.9	±3.7	22.6	±3.7	0.7	±0.2	13.3	±4.5	13.3	±4.6	0.2	±0.2	0.2	±0.2
		(16.0	34.8)	(17.0	35.6)	(0.3	1.0)	(7.5	28.3)	(7.5	28.5)	(0.0	0.6)	(0.0	0.6)
M2	37 PMs	22.2	±3.5	22.6	±3.5	0.5	0.2	13.3	±4.2	13.4	±4.2	0.3	±0.2	0.3	±0.2
		(16.4	33.3)	(17.4	33.8)	(0.2	1.0)	(7.5	26.2)	(7.6	26.4)	(0.1	0.6)	(0.1	0.6)
M3	37 PMs	22.3	±2.7	23.2	±2.7	0.9	0.1	13.2	±3.7	14.1	±3.6	0.4	±0.2	0.4	±0.2
		(17.1	30.7)	(18.2	31.7)	(0.8	1.3)	(7.2	23.3)	(8.3	23.9)	(0.1	0.8)	(0.1	0.8)
M4	37 PMs	22.4	±2.8	23.3	±2.7	0.9	0.1	13.2	±3.7	14.1	±3.6	0.4	±0.2	0.4	±0.2
		(17.0	30.9)	(18.1	31.9)	(0.8	1.2)	(8.5	23.5)	(9.4	24.1)	(0.1	0.8)	(0.1	0.7)
M5	37 PMs	22.4	±3.1	23.3	±3.0	0.9	0.2	12.7	±4.0	13.5	±4.0	0.3	±0.2	0.4	±0.2
		(16.8	32.5)	(18.0	33.5)	(0.1	1.2)	(6.6	25.5)	(7.6	26.1)	(0.1	0.9)	(0.1	0.9)
M6	37 PMs	22.6	±3.4	22.8	±3.3	0.2	0.2	13.2	±4.0	13.2	±4.0	0.4	±0.2	0.4	±0.2
		(14.5	32.0)	(15.3	32.4)	(0.0	0.8)	(4.6	25.0)	(4.7	24.9)	(0.1	0.9)	(0.1	0.9)
M7	37 PMs	22.5	±2.7	23.0	±2.7	0.5	0.2	12.8	±4.3	13.2	±4.7	0.3	±0.4	0.3	±0.4
		(17.8	31.0)	(18.4	31.8)	(0.0	1.1)	(0.0	23.6)	(0.0	23.9)	(0.1	2.7)	(0.0	0.9)
M8	37 PMs	21.7	±2.7	22.6	±2.6	0.9	0.1	12.6	±4.0	13.5	±3.9	0.3	±0.2	0.4	±0.2
		(16.7	29.4)	(17.9	30.5)	(0.6	1.3)	(6.5	22.1)	(7.3	22.7)	(0.1	0.8)	(0.1	0.7)
M9	37 PMs	20.1	±2.7	23.7	±2.6	3.1	0.9	10.5	±4.0	14.7	±3.6	0.4	±0.2	0.3	±0.2
		(16.0	28.9)	(18.6	31.6)	(0.8	5.2)	(3.4	21.7)	(7.3	23.9)	(0.1	0.9)	(0.0	0.7)
M10	37 PMs	20.3	±3.2	24.2	±3.2	3.5	1.0	11.6	±4.4	15.2	±3.9	0.4	±0.2	0.3	±0.2
		(15.4	32.4)	(18.9	35.7)	(0.9	5.8)	(1.6	25.5)	(7.6	28.4)	(0.1	1.0)	(0.0	0.7)
M11	37 PMs	22.3	±3.0	23.2	±2.9	0.9	0.1	12.7	±4.1	13.5	±4.0	0.3	±0.2	0.4	±0.2
		(16.7	32.6)	(17.9	33.7)	(0.7	1.2)	(6.5	25.6)	(7.6	26.2)	(0.1	0.8)	(0.1	0.7)
M12	37 PMs	20.4	±3.3	24.0	±3.4	4.0	1.2	11.9	±4.4	15.4	±3.9	0.4	±0.2	0.2	±0.2
		(15.6	32.9)	(18.7	36.4)	(2.0	8.2)	(3.1	26.0)	(8.4	29.1)	(0.1	0.9)	(0.0	0.6)

Shown are the median, standard deviation, minimum and maximum (in brackets) of *t*_rec_, the *leukopenia time* *t*_leu_ (the number of days with WBC count ≤1 *G*/*L*) and *nadir* for D123 and D135 schedules. The first two rows show values from two independent clinical studies that serve as a comparison. The second part of the table shows prediction results. Predictions were calculated with PMs from our clinical data with underlying mathematical models M1–M12. Model M5 explained well the outcome of schedule D135, but showed a significant mismatch of more than three days for schedule D123. The predictions using the extended model M10 were better for schedule D123. See also Fig 4e and 4f for an illustrated comparison between M5 and M10. Model M9 and M12 were also promising, but we focused on M10 applying Ockam’s razor.

### Schedule timing

**Fig 5:**
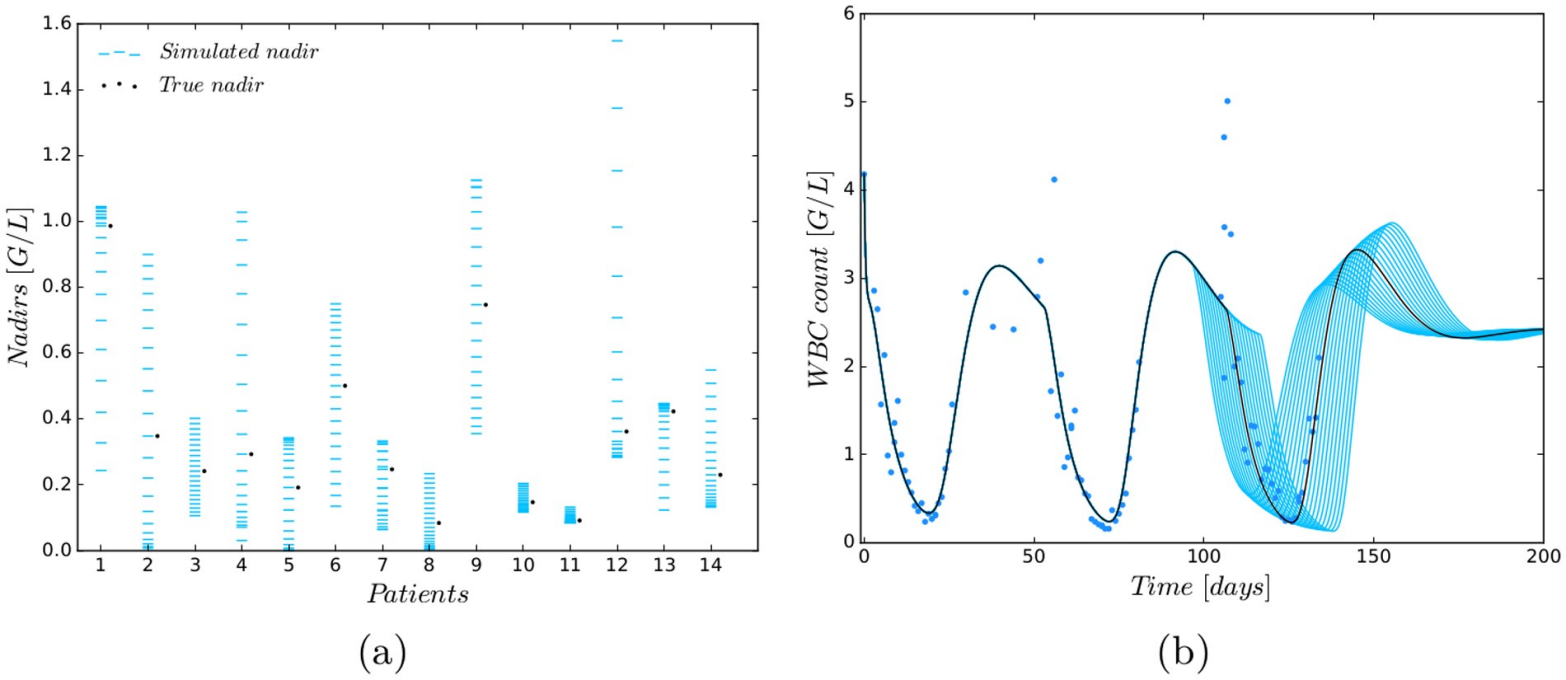
Analysing the influence of treatment timing on nadir values. (**a**) Simulation study in which 20 simulated nadirs were compared with the true nadir of the last CC for the 14 patients who have more than one CC. The simulated nadirs were computed by using the patient’s PM (second row of Table 3) and varying the start of the last CC daily with the maximal starting variation of 10 days earlier or later. (**b**) Exemplary variation of the CC start for one patient. An earlier (later) start results in a larger (lower) nadir.

Similar to previous simulation studies dealing with varying and shortening cycle duration and finding the optimal number and timing of G-CSF administrations to reduce myelosuppression [52–54], we analysed the impact of different treatment starts of the last CCs with respect to obtained nadir values. A comparison to the clinically observed nadir values indicated a large potential for clinical improvement, i.e., a higher nadir value due to a different treatment timing (see Fig 5a). Fig 5b exemplarily shows the WBC dynamics for different treatment timings. Earlier (later) starts resulted in sequentially higher (lower) nadir values.

## Discussion

High-density WBC counts from 23 AML patients were collected and used to personalise 12 mathematical models and analyse their prediction accuracy with respect to different modelling hypotheses and treatment schedules. The high prediction accuracies of the PMs, especially around the nadir, confirm previous claims [4, 24] that the general approach of in-silico studies can be used for clinical decision support. As clinical decision support we understand tools which help physicians to monitor and predict WBC dynamics and the duration and grade of myelosuppression. In combination with clinical expertise on the impact of schedules on relapse probabilities and their small scope determining the start of the next cycle due to subjective experience and the patients fitness, this might have an important clinical impact via altered treatment schedules which might eventually result in decreased depth and duration of myelosuppression.

Current drawbacks are the high model uncertainty, if insufficient information is available. This makes precise and reliable predictions difficult (compare Fig 3c and 3d). Furthermore, the lack of leukemic cell dynamics and the validation of the PMs based on one specific chemotherapy schedule might lead to not appropriate models concerning an optimisation of Ara-C dosage.

Comparing the estimated parameter values with published values, the estimated baseline value for the WBCs is within the normal human WBC range of 4.5−10 *G*/*L* but reduced by 2 *G*/*L* compared to published baseline values for the Friberg model being in the range of 7 to 7.8 *G*/*L* [12]. The mean maturation time of 128 h for M3 using the corrected formula [17] is reasonable and fits into the range of previously published values [55]. The estimated *γ* values are roughly two to three times higher compared to published values for the reason that we only use one transition compartment. The *γ* values for M2, containing three transition compartments, are in the same range then published values. The decrease of the slope parameter value from one to two PD effects occurs as the effect of Ara-C is distributed on two different sites of action. During the parameter estimation we observed for some CCs correlations (> 0.9) between *γ* and *k*_tr_ and between *γ* and slope. But these correlations had no influence on the parameter identifiability. Further, it was shown that under certain assumptions, which we fulfilled (*k*_prol_ = *k*_tr_), the Friberg model is structurally globally identifiable [55]. In future studies we propose to use global design measures from [56] to provide treatment schedules reducing global parameter sensitivity and undesired parameter correlation.

We showed that an analysis based on a fixed chemotherapy schedule cannot discriminate between different modelling hypotheses. The agglomerative nature of the mathematical models leads to a choice of model parameters that is not only personalised to the patient, but also to the applied schedule. Therefore, we used different schedules for personalisation and prediction to overcome this problem and to allow discrimination of the models. This approach allowed us to distinguish between the modelling hypotheses implemented in models M5–M12 and enabled us to find the suitable model assumption considered in M9, M10, and M12. In our opinion this procedure should be routinely applied, preferably using high density WBC counts for different schedules in the same patients. As an alternative to such a tedious clinical study we suggest to use average *t*_rec_ values as a discrimination criterion for competing models.

Comparing the *t*_rec_ values for D123 and D135 treatments from the PMs with our clinical data and the AMLSG 07-04 trial in Table 4 and S2 Table implies, that model M10 (based on Ockam’s razor in comparison to M9 and M12) is the best candidate among M1–M12 for future work on the simulation and optimisation of intermediate to high-dose Ara-C treatment schedules.

The 1 day shift in *t*_*rec*_ values between our clinical data and the AMLSG 07-04 trial can be explained by the age difference between patients in our clinical data (median 62 and 57 years for D123 and D135, respectively) and the subdata of the AMLSG 07-04 trial (median of all patients in the trial 49 years) and a related statistical analysis: Jaramillo *et al*. [46] found in a multivariable analysis a significantly longer WBC recovery for older patients (hazard ratio of a 10-year age difference, 0.89; P = 0.001) [46] and a significantly shorter WBC recovery for patients receiving D123 compared to the reference group D135 (hazard ratio, 1.94; P < 0.0001) [46] which coincides well with our findings.

Regarding the PK model, no published compartment models for high-dose Ara-C are available. Comparing our model with published low-dose Ara-C models, we showed that the published models do not reach the measured maximum Ara-C concentrations from high-dose schedules (see Fig 2) so that we rely on our derived model. As we logarithmised the collected Ara-C concentrations, lower values became more important during parameter estimation such that our fitted PK model slightly underpredicts the highest Ara-C concentrations (see Fig 2). Nevertheless, our model achieves higher values compared to the models from [20] and [47] providing a more reasonable PK behaviour of Ara-C. We estimated a smaller central volume leading to a reduced clearance activity derived from an almost equivalently estimated elimination rate constant value (Table 2). The distribution rate constants differ by a factor of 2 to 2.5 and the peripheral volume by a factor of almost 10. Future PK studies for high-dose Ara-C can be used for model verification or updating our model parameters. In a simulation study we analysed the influence of constructed PK variability on the WBC recovery time for models M5 and M10. We showed that model M10 is more sensitive to varied PK dynamics and reflects clinical findings more accurately, i.e. that the standard and dense treatment plans result in significantly different WBC recovery times. M5 was not able to match the clinical results. A critical part of the study is the constructed IIV. In Krogh *et al*. [20] IIV was analysed for low-dose Ara-C schedules. We used the published values as exemplary IIV values within our simulation study. Obviously, the results should be treated with care as IIV is related to the underlying study, treatment, model and population and thus cannot be applied to other studies in general. However, the IIV impacted only a small part of our sensitivity analysis and we do not expect qualitative changes for updated IIV values.

Summarising, we extended the gold-standard model for myelosuppression [12] to the most important component in consolidation therapy [2, 25], Ara-C, and showed that one modelling assumption was important for a faster WBC count recovery for D123 schedules. In models M9, M10, and M12 we assumed that the Ara-C concentration has a direct impact on the proliferation speed. As stated above, such a modelling assumption has an agglomerative nature and the underlying physiological processes are still unknown.

We speculate that the increased number of cell deaths following chemotherapy might play a role. Cell deaths induce phagocytosis and macrophage activation, which in turn might increase G-CSF secretion and hence proliferation speed [57] resulting in a positive *circulus vitiosus*. This might also be one explanation for the observation that G-CSF levels in neutropenia are increased after chemotherapy compared to primary diseases [58]. Independent from the underlying phyiological process, the dense treatment schedule D123 profits more than the standard plan D135 from the induced dynamics with respect to WBC recovery times as discussed in S4 and S5 Figs.

Future G-CSF concentration measurements for AML patients during consolidation cycles of D123 and D135 treatments and a comparison of our extended models with Quartino’s [30] integrated G-CSF-myelosuppression model or more sophisticated models from quantitative systems pharmacology [54] may shed light on these speculations.

## Supporting information

S1 Appendix (PDF)

S1 File NONMEMtableAMLcyclewise181210.csv. (CSV)

S2 File NONMEMtableAMLpatientwise181210.csv. (CSV)

S3 File kern1997_3mg.py. (PY)

S1 Table Terminology and potentially confusing synonyms. Expressions that are also used in the manuscript are in *italic*.
(PDF)

S2 Table Comparison of model predictions for low-dose treatment schedules. As in Table 4, predicted *nadir* values for different treatment schedules are shown, based on underlying mathematical models M1–M12. Shown are the values of median, standard deviation, minimum and maximum (in brackets) for two low-dose schedules. Both assume a continuous infusion throughout days 1 to 5, with either 100 *mg*/*m*^2^ or 400 *mg*/*m*^2^ Ara-C per day. No clinical observations are available to compare these predictions, but they give additional insight on the possibility to discriminate models M1–M12 and a general trend showing that for 100 *mg*/*m*^2^ per day despite of M7-M9 almost all nadir values are above 1 *G*/*L*. The nadir values for the low-dose infusion with 400 *mg*/*m*^2^ Ara-C per day are in the same range compared to the results of the high-dose schedules (Two further personalised cycles were excluded because for some models no recovery after chemotherapy was observed). The simulated nadirs above 1 *G*/*L* for the low-dose schedule (100 *mg*/*m*^2^) reflect the lower toxic effects represented by required hospitalisation due to fever and neutropenia and platelet transfusions compared to the low-dose (400 *mg*/*m*^2^) and high-dose schedules explored in [3]. As M7-M9 are not able to reflect the lower toxic effects through higher nadir values, the simulation study serves as an indicator that the secondary effect of Ara-C may not be an Ara-C induced reduction of the transition rate.
(PDF)

S3 TableModel constants, patient-specific constants, and units of model parameters.The values were used to obtain personalised mathematical models. The constants were determined from published data [39] and applied to all patients. To shorten notation we also used $c_{V}=\frac{1}{V_{c}\;MM_{\text{cyt}}}$. The patient-specific infusion times and dosages that define a treatment schedule were modified for simulation and optimisation of different schedules. The range shows minimum and maximum values of all considered data in the clinical study.(PDF)

S4 TableObjectives (final objective function values from FOCEi method (OBJ), population predicted $t_{rec}^{123}$ and $t_{rec}^{135}$ values), parameter and coefficient of variation (CV) estimates with relative standard errors (RSE) from nonlinear mixed-effects modelling of models M3 and M10 with initial condition approach I1 (with I1).(PDF)

S5 TableObjectives (final objective function values from FOCEi method (OBJ), population predicted $t_{rec}^{123}$ and $t_{rec}^{135}$ values), parameter and coefficient of variation (CV) estimates with relative standard errors (RSE) from nonlinear mixed-effects modelling of model from Stiehl *et al*. [8].(PDF)

S1 Fig Goodness-of-fit plot for all but three (because of WBC counts greater 1) measured and calculated *t*_*rec*_ values for M10 after model personalisation for each consolidation cycle. The 39 measured *t*_rec_ values are slightly higher due to the coarser measurement grid.
(PDF)

S2 Fig Cross-validation of predicted white blood cell (WBC) counts from personalised models (PMs) M1-M12 and measured WBC counts for six patients treated with D135. All but one cycle were used for personalisation and the last cycle for cross-validation. For patients (a)-(e) the PMs can predict the WBC count decrease after Ara-C administration in the last cycle where models M10 and M12 have a slower WBC recovery than M5. For patient (f) the WBC recovery from the PMs starts to early compared to the measured WBC counts.
(PDF)

S3 Fig Cross-validation of predicted white blood cell (WBC) counts from personalised models (PMs) M1-M12 and measured WBC counts for five patients (a)-(e) treated with d135 and one patient (f) treated with D12. The PMs provide good predictions for patient (a) and (f) but show mismatches in recovery times and nadir values for patients (b)-(e).
(PDF)

S4 Fig Comparing personalised mathematical models (PMs) M5 and M10 for D123 and D135 schedules (exemplary patient I). (PDF)

S5 Fig Comparing personalised mathematical models (PMs) M5 and M10 for D123 and D135 schedules (exemplary patient II). (PDF)

S6 Fig Comparing personalised mathematical models (PMs) M10 and M12 for D123 and D135 schedules (exemplary patient I). (PDF)

S7 Fig Visual predictive checks (VPCs), derived by 1000 simulations, for leukocytes [*G*/*L*] versus time [*days*] starting with the first measurement before dosing for model M3 (a) and M10 (with I1) (b). Blue circles are the measured WBC counts of 23 AML patients described in section *Clinical Data & Personalisation*. One measurement was taken at timepoint 88.98 [days] with the value 7.18 [G/L] which is not shown in the VPCs. Red lines show the median (solid) and 5th and 95th percentiles (dashed) of measurements. The shaded areas represent the 95% confidence intervals around the 5th (blue), 50th (red) and 95th (blue) simulated percentiles of the model predictions. Regarding the VPCs, model M3 and M10 have an almost equivalent prediction accuracy. The 50% percentiles of measurements and model predictions perfectly overlap, thus supporting our individually based results from Table 3. The same applies to the start of the 5% and 95% percentiles until the nadir. After the nadir the 5% and 95% percentiles of the model predictions recover slightly faster/slower compared to the measurements. At day 30 the percentiles of measurements and model predictions coincide again.
(PDF)

S8 Fig Simulation study analysing the sensitivity of model M5 and M10 on inter-individual PK variability (IIV) when schedules D123 and D135 are applied. (**a**) As Fig 2, but with 500 simulations of our fitted two-compartment PK model with IIV on the clearance and the central volume. (**b**) Recovery times (*t*_*rec*_) from 500 simulations each of models M3 and M10 (with I1) applying schedules D123 and D135 with inter-individual variability given as coefficient of variation (CV) on PK parameters clearance (45%) and central volume (70%). Red lines within the boxes are the medians, the upper and lower box limits are the first (Q1) and third quartiles (Q3) of the data. The lower whiskers will extend to the first *t*_*rec*_ values greater than the first quartiles minus the 1.5-times the interquartile ranges (IQR) (*Q*1 − 1.5 * *IQR*). Equivalently, the upper whiskers will extend to last *t*_*rec*_ values less than *Q*3 + 1.5 * *IQR*. Beyond the whiskers, data are considered as outliers and are plotted as individual points (+). The simulation study revealed that model M10 was more sensitive to different high-dose Ara-C treatment schedules compared to model M3 despite the high inter-individual PK variability.
(PDF)
