# Supplementary material for: Mathematical models for cytarabine-derived myelosuppression in acute myeloid leukaemia
Source: PLoS One. 2019 Jul 1;14(7):e0204540. doi: 10.1371/journal.pone.0204540 (PMC6602180; doi:10.1371/journal.pone.0204540)
Supplement: S1 Appendix — (PDF) [file pone.0204540.s001.pdf]

S1 Appendix

Section 1 introduces parameter estimation, uncertainty quantification, how we define the accuracy of mathematical models and the nonlinear mixed-effects analysis.

1 Mathematical Methods

Parameter Estimation

To personalise mathematical models we solved nonlinear least-squares problems of the type

$$\min_{p,x} \quad \frac{1}{2}\sum_{i=1}^{m}\frac{(\eta_i - x_{\text{ma}}(t_i))^2}{\sigma_i^2} + \alpha \left\| x_{\text{pr}}(t_0) - B_{\text{bm}} \right\|_2^2 + \sum_{i=1}^{n_{\text{tr}}} \alpha \left\| x_{\text{tr},i}(t_0) - B_{\text{bm}} \right\|_2^2 \quad (19)$$

$$\text{s.t.} \quad \dot{x}(t) = f(x(t), u(t), p), \quad x(t_0) = x_0(p) \quad (20)$$

with a vector $p = (B, k_{\text{tr}}, \gamma, \text{slope}, x_{\text{pr}}(t_0), x_{\text{tr}}(t_0), x_{\text{ma}}(t_0))$ of model parameters, dynamics (1) given by a $f(\cdot)$ where we write $x_{\text{tr}} = (x_{\text{tr},1}, \ldots, x_{\text{tr},n_{\text{tr}}})$, initial conditions $x_0(p)$ that are chosen according to I1 for M1–M3, I2 for M4, and I3 for M5–M12, and a penalisation factor $\alpha = 1/2500$ (note that the penalty terms vanish for strategies I1 and I2 as the initial conditions are fixed to B_{bm}). The variances σ_i were chosen as $\sigma_i = 0.3 + 0.3\eta_i$ defining additive plus proportional residual variabilities with constant variances [51]. This formula was chosen after trial and error with different scalings and visual assessment of results, as we did not estimate the variances σ_i during the individual parameter estimations. Among the candidates were also logarithmic functions which are often used in the literature. The WBC count data vector η has m entries and depends on the particular data from Study I used for personalisation.

The parameter estimation problems (19) were solved with a multiple shooting based Gauß–Newton algorithm coded in the PAREMERA software and an adaptive, error–controlled backward differentiation formulae (BDF) method for integration coded in the software DAESOL, both included in the experimental design package VPLAN [50] developed at the University of Heidelberg. The same integrator was used for all individual predictions (simulations) in this paper. Note that the Gauß–Newton algorithm, like all Newton-type algorithms, only converges to a local minimum. Published parameter estimates served as starting values for the optimization problems. We also tested different starting values with whom the parameter estimation problems converged to the same minima.

Uncertainty Quantification

In Fig 3c–d we visualised uncertainty tubes of PMs by means of 1000 Monte Carlo simulations to indicate the propagated probability density function. Each simulation was performed with a set of parameters $p^s = (B, \gamma, k_{\text{tr}}, \text{slope}, x_{\text{pr}}(t_0), x_{\text{tr}}(t_0), x_{\text{ma}}(t_0))$ sampled from the multivariate normal distribution $\mathcal{N}_7(p^*, C^*)$. The vector p^* was the nominal solution of (19) and C^* the corresponding variance-covariance matrix provided by the software package VPLAN. A detailed description of the computation of the variance-covariance matrix C^* can be found in [50].

Accuracy of mathematical models

InTable 3 we evaluated the accuracy of personalised mathematical models PMs using the root mean squared error

$$\mathrm{RMSE} = \sqrt{\frac{1}{m}\sum_{i=1}^{m}(\eta_i - x_{\mathrm{ma}}(t_i))^2} \tag{21}$$

where $x_{\mathrm{ma}}(\cdot)$ is part of the solution of the estimation problem (19).

Nonlinear mixed-effects modelling

To qualitatively confirm that the extensions in models M9, M10, and M12 cover secondary effects of Ara-C we performed a nonlinear mixed-effects modelling approach on the 42 consolidation cycles for the most relevant models M3 and M10 with initial value condition I1. We were led by previously published NLME models for myelosuppression, used optimisation algorithms and considered objective function values (due to comparing nested models), plausibility of the parameter estimates, the magnitude of their relative standard errors and the related parameter identifiability for model development. A comparison between the results of the first-order conditional estimation (FOCE) and the first-order (FO) method indicated that the set of population parameter values from the FO method and only adapting dosing schemes with respect to body surface area and age is not sufficient to provide high individual model accuracies. The FO method resulted in high inter-individual variabilities for k_{tr}, slope and γ and the objective function value was 62.80 compared to -68.78 using the FOCE method.

Applying initial value approach I3 with prior knowledge in NONMEM resulted in parameter estimates achieving 15% reduction of the objective value, but with the drawback of high standard errors. Thus, we applied the steady state approach I1 for the reason that our main focus is on predicting leukopenia. Finally, inter–individual variability (IIV) was assumed to be log-normally distributed and residual variability was estimated using a proportional error model. Parameter estimation was performed in NONMEM (version 7.4, first-order conditional estimation method with interaction as used in previous publications) in combination with PsN software (version 4.4.0; Uppsala Pharmacometrics, Uppsala, Sweden). Data set preparation was performed in Python (version 2.7.6) and analysis of the results was performed in R (version 3.4.4; R Project for Statistical Computing, Vienna, Austria) including the xpose4 package (version 4.6.1) for generating visual predictive checks.

To analyse the effect of the PK variability on the different modelling hypotheses, we performed 500 simulations each using NONMEM for the nonlinear mixed-effects models of M3 and M10 (with I1) applying schedules D123 and D135 with fixed population parameter values for B, slope, k_{tr}, and γ (see S4 Table) and inter-individual PK variability on clearance and central volume. The population parameter values of the PK model were derived from the naive pooling approach (19) and the inter-individual variability was taken from [20]. A detailed description is given below of S8 Fig.
