# Supplementary material for: Mathematical models for cytarabine-derived myelosuppression in acute myeloid leukaemia
Source: PLoS One. 2019 Jul 1;14(7):e0204540. doi: 10.1371/journal.pone.0204540 (PMC6602180; doi:10.1371/journal.pone.0204540)
Supplement: S1 Table — Expressions that are also used in the manuscript are in italic. (PDF) [file pone.0204540.s005.pdf]

S1 Table. Terminology and potentially confusing synonyms.

Expression	Synonyms or related concepts
bone marrow precursor WBC	proliferating and maturing cells, *myeloid stem cells*, cells in *proliferating and transition compartments*, *progenitor cells*
cross-validation	*estimation* and *prediction*: comparing measured data to predictions of *personalised models* derived from different data
low WBC count	*leukopenia*, granuolocytopenia, neutropenia
initial conditions	*initial values*
measured	observed
model parameter	variable number in the differential equations
modelling assumptions	hypotheses on what physiological effects are relevant to represent the most important dynamics
baseline WBC count	WBC steady state value, normal WBC count
personalised	estimated, fitted, calibrated, after parameter estimation
personalised mathematical model	personalised dynamic model, parameterised model, fitted/estimated/calibrated (mathematical) model
schedule	treatment, regimen, control
slower WBC recovery	leukopenia with longer duration, prolonged leukopenia, delay in WBC recovery
to assume	to conjecture, to claim, to 0
to optimise	to calculate the treatment schedule which is locally optimal for the personalised mathematical model
to predict	to simulate, to forecast, to solve differential equations

Expressions that are also used in the manuscript are in *italic*.
