# Supplementary material for: Mathematical models for cytarabine-derived myelosuppression in acute myeloid leukaemia
Source: PLoS One. 2019 Jul 1;14(7):e0204540. doi: 10.1371/journal.pone.0204540 (PMC6602180; doi:10.1371/journal.pone.0204540)
Supplement: S2 Table — As in Table 4, predicted nadir values for different treatment schedules are shown, based on underlying mathematical models M1–M12. Shown are the values of median, standard deviation, minimum and maximum (in brackets) for two low-dose schedules. Both assume a continuous infusion throughout days 1 to 5, with either 100 mg/m2 or 400 mg/m2 Ara-C per day. No clinical observations are available to compare these predictions, but they give additional insight on the possibility to discriminate models M1–M12 and a general trend showing that for 100 mg/m2 per day despite of M7-M9 almost all nadir values are above 1 G/L. The nadir values for the low-dose infusion with 400 mg/m2 Ara-C per day are in the same range compared to the results of the high-dose schedules (Two further personalised cycles were excluded because for some models no recovery after chemotherapy was observed). The simulated nadirs above 1 G/L for the low-dose schedule (100 mg/m2) reflect the lower toxic effects represented by required hospitalisation due to fever and neutropenia and platelet transfusions compared to the low-dose (400 mg/m2) and high-dose schedules explored in [3]. As M7-M9 are not able to reflect the lower toxic effects through higher nadir values, the simulation study serves as an indicator that the secondary effect of Ara-C may not be an Ara-C induced reduction of the transition rate. (PDF) [file pone.0204540.s006.pdf]

S2 Table. Comparison of model predictions for low-dose treatment schedules.

		nadir			
**Model**	**Group**	**D1-5 100**		**D1-5 400**	
M1	35 PMs	1.6	±0.8	0.3	±0.2
		(0.0	3.6)	(0.0	0.8)
M2	35 PMs	1.7	±0.8	0.3	±0.2
		(0.5	3.7)	(0.1	1.0)
M3	35 PMs	1.8	±0.9	0.4	±0.2
		(0.8	4.3)	(0.2	1.1)
M4	35 PMs	1.7	±1.0	0.4	±0.2
		(0.8	4.9)	(0.2	1.1)
M5	35 PMs	1.7	±1.1	0.4	±0.2
		(0.7	5.5)	(0.2	1.0)
M6	35 PMs	1.5	±1.1	0.4	±0.3
		(0.9	5.9)	(0.2	1.2)
M7	35 PMs	0.5	±0.5	0.1	±0.3
		(0.1	3.4)	(0.0	1.0)
M8	35 PMs	0.1	±0.1	0.1	±0.1
		(0.0	0.4)	(0.0	0.4)
M9	35 PMs	0.7	±0.4	0.2	±0.1
		(0.1	1.6)	(0.0	0.5)
M10	35 PMs	2.1	±1.3	0.6	±0.4
		(0.6	5.8)	(0.1	1.8)
M11	35 PMs	1.6	±1.1	0.4	±0.2
		(0.9	5.5)	(0.2	1.0)
M12	35 PMs	2.4	±1.2	0.7	±0.5
		(0.8	5.8)	(0.1	2.1)

As in Table 4, predicted *nadir* values for different treatment schedules are shown, based on underlying mathematical models M1–M12. Shown are the values of median, standard deviation, minimum and maximum (in brackets) for two low-dose schedules. Both assume a continuous infusion throughout days 1 to 5, with either 100 mg/m^2 or 400 mg/m^2 Ara-C per day. No clinical observations are available to compare these predictions, but they give additional insight on the possibility to discriminate models M1–M12 and a general trend showing that for 100 mg/m^2 per day despite of M7-M9 almost all nadir values are above 1 G/L. The nadir values for the low-dose infusion with 400 mg/m^2 Ara-C per day are in the same range compared to the results of the high-dose schedules (Two further personalised cycles were excluded because for some models no recovery after chemotherapy was observed). The simulated nadirs above 1 G/L for the low-dose schedule (100 mg/m^2) reflect the lower toxic effects represented by required hospitalisation due to fever and neutropenia and platelet transfusions compared to the low-dose (400 mg/m^2) and high-dose schedules explored in [3]. As M7-M9 are not able to reflect the lower toxic effects through higher nadir values, the simulation study serves as an indicator that the secondary effect of Ara-C may not be an Ara-C induced reduction of the transition rate.
