# Supplementary material for: Mathematical models for cytarabine-derived myelosuppression in acute myeloid leukaemia
Source: PLoS One. 2019 Jul 1;14(7):e0204540. doi: 10.1371/journal.pone.0204540 (PMC6602180; doi:10.1371/journal.pone.0204540)
Supplement: S3 Table — The values were used to obtain personalised mathematical models. The constants were determined from published data [39] and applied to all patients. To shorten notation we also used cV=1VcMMcyt. The patient-specific infusion times and dosages that define a treatment schedule were modified for simulation and optimisation of different schedules. The range shows minimum and maximum values of all considered data in the clinical study. (PDF) [file pone.0204540.s007.pdf]

S3 Table. Model constants, patient-specific constants, and units of model parameters.

**Constant**	**Unit**	**Value**
Ara-C rate elimination k_{10}	$1/day$	98.64
Ara-C rate distribution k_{12}	$1/day$	2.69
Ara-C rate distribution k_{21}	$1/day$	1.29
Molecular Mass MM_{cyt}	g/mol	243.217
Volume of central compartment V_c	L	37.33
Death rate k_{ma}	$1/day$	2.3765
**Patient-Specific**	**Unit**	**Range**
Body Surface Area BSA	m^2	$[1.61, 2.07]$
Infusion duration	day	$3/24$
Ara-C dosage	g/m^2	$[1, 3]$

**Model parameter**		**Unit**
k_{tr}	Transition rate	$1/day$
γ	Feedback exponent	–
slope	Pharmacodynamics	$L/\mu mol$
B	Baseline WBC count	$10^9/L$
$x_{\mathrm{pr}}(0)$	Initial value	$10^9/L$
$x_{\mathrm{tr}}(0)$	n_{tr} initial values	$10^9/L$
$x_{\mathrm{ma}}(0)$	Initial value	$10^9/L$

The values were used to obtain personalised mathematical models. The constants were determined from published data [39] and applied to all patients. To shorten notation we also used $c_V = \frac{1}{V_c\ MM_{\mathrm{cyt}}}$. The patient-specific infusion times and dosages that define a treatment schedule were modified for simulation and optimisation of different schedules. The range shows minimum and maximum values of all considered data in the clinical study.
