# Supplementary material for: Mathematical models for cytarabine-derived myelosuppression in acute myeloid leukaemia
Source: PLoS One. 2019 Jul 1;14(7):e0204540. doi: 10.1371/journal.pone.0204540 (PMC6602180; doi:10.1371/journal.pone.0204540)
Supplement: S4 Table — (PDF) [file pone.0204540.s008.pdf]

S4 Table. Objectives (final objective function values from FOCEi method (OBJ), population predicted t_{rec}^{123} and t_{rec}^{135} values), parameter and coefficient of variation (CV) estimates with relative standard errors (RSE) from nonlinear mixed-effects modelling of models M3 and M10 with initial condition approach I1.

	**M3**	**M10 (with I1)**
**Objectives**		
t_{rec}^{123}	22.09	20.35
t_{rec}^{135}	22.98	23.96
Final OBJ	-353.94	-348.49
**Fixed Effects (RSE%)**		
B	5.3248 (6)	5.1939 (9)
k_{tr}	0.18694(5)	0.19383(4)
γ	0.48587(9)	0.46885(5)
slope	8.5915 (9)	5.5101 (7)
**inter–individual Variability CV%(RSE%)**		
B	35.2(11)	34.9(10)
k_{tr}	21.0(17)	21.7(16)
γ	32.4(24)	36.6(23)
slope	38.9(16)	34.3(14)
**Residual Error (CV%)**		
Proportional	0.109(9)	0.109(10)

The estimated parameters are approximately in the same range as published values which considered neutrophils and not leukocytes [22, 31]. This is true for B and its inter–individual variability (IIV). A comprehensive discussion of k_{tr} and the related mean maturation time is given in the main text. The parameter slope cannot be compared as we present the first study with high-dose Ara-C. The estimated γ value is roughly two to three times higher compared to published values for the reason that we only use one transition compartment. The γ values for M2, containing three transition compartments, are in the same range then published values.
