# Supplementary material for: Mathematical models for cytarabine-derived myelosuppression in acute myeloid leukaemia
Source: PLoS One. 2019 Jul 1;14(7):e0204540. doi: 10.1371/journal.pone.0204540 (PMC6602180; doi:10.1371/journal.pone.0204540)
Supplement: S5 Table — (PDF) [file pone.0204540.s009.pdf]

S5 Table. Objectives (final objective function values from FOCEi method (OBJ), population predicted t_{rec}^{123} and t_{rec}^{135} values), parameter and coefficient of variation (CV) estimates with relative standard errors (RSE) from nonlinear mixed-effects modelling of model from Stiehl *et al.* [8].

	**Stiehl2018** [8]
**Objectives**	
t_{rec}^{123}	25.16
t_{rec}^{135}	25.68
Final OBJ	-399.89
**Fixed Effects (RSE%)**	
a_1	0.5929(13)
a_2	0.0003(89)
p_1	1.9880(80)
p_2	0.1971 (8)
k	0.0337(75)
slope	0.9270(74)
**IIV CV%(RSE%)**	
a_1	1.8 (176)
a_2	32.4(47222)
p_1	44.4 (195)
p_2	28.7 (136)
k	32.1 (19)
slope	33.4 (60)
**Residual Error (CV%)**	
Proportional	0.106(5)

We analysed the three compartment model from Stiehl *et al.* [8] in which, except for the last compartment, each compartment contains parameters for the fraction of self-renewal (a_1, a_2) and for differentiation (p_1, p_2). Similar to the general mathematical model (1) the pharmacodynamic effect E is applied to the proliferating cells c_1, complementing the state equation of c_1 by the term $-Ea_1s(t)p_1c_1$. As it is not exactly known at which maturation step the ability of self-renewal gets lost, the values of the fractions of self-renewal $a_i,\ i = 1, \ldots, n_{tr}$ can help specifying the step. As we only consider one transition compartment it turns out that almost only the stem cells perform self-renewal as the fixed effect parameter value of a_2 is close to zero. After the chemotherapy the cells in the transition compartment draw their whole ability on differentiation such that the WBC recovery is speeded up and homeostasis is reached again as soon as possible. Due to the deviation of the recovery times with clinical findings and the large relative standard errors, we did not use the model for further investigations.
