# Supplementary material for: Mathematical models for cytarabine-derived myelosuppression in acute myeloid leukaemia
Source: PLoS One. 2019 Jul 1;14(7):e0204540. doi: 10.1371/journal.pone.0204540 (PMC6602180; doi:10.1371/journal.pone.0204540)
Supplement: S1 Fig — The 39 measured trec values are slightly higher due to the coarser measurement grid. (PDF) [file pone.0204540.s010.pdf]

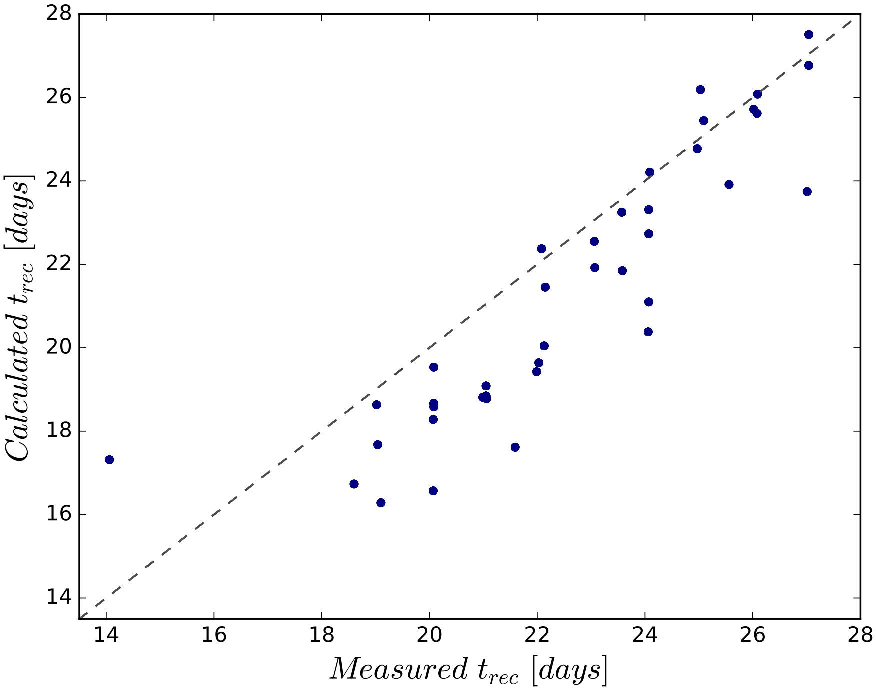

S1 Fig. Goodness-of-fit plot for all but three (because of WBC counts greater 1) measured and calculated t_{rec} values for M10 after model personalisation for each consolidation cycle. The 39 measured t_{rec} values are slightly higher due to the coarser measurement grid.
