# Supplementary material for: Mathematical models for cytarabine-derived myelosuppression in acute myeloid leukaemia
Source: PLoS One. 2019 Jul 1;14(7):e0204540. doi: 10.1371/journal.pone.0204540 (PMC6602180; doi:10.1371/journal.pone.0204540)
Supplement: S2 Fig — All but one cycle were used for personalisation and the last cycle for cross-validation. For patients (a)-(e) the PMs can predict the WBC count decrease after Ara-C administration in the last cycle where models M10 and M12 have a slower WBC recovery than M5. For patient (f) the WBC recovery from the PMs starts to early compared to the measured WBC counts. (PDF) [file pone.0204540.s011.pdf]

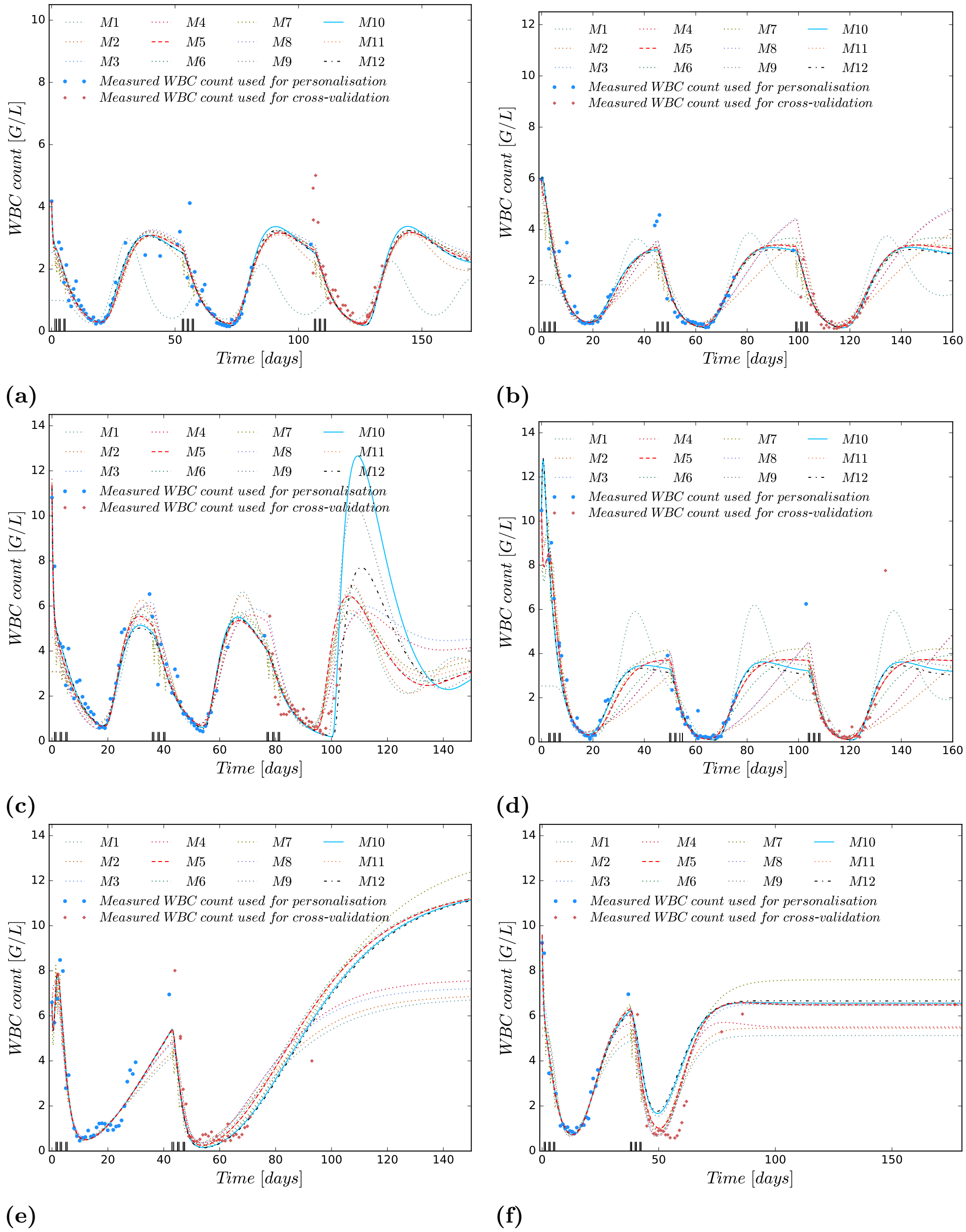

S2 Fig. Cross-validation of predicted white blood cell (WBC) counts from personalised models (PMs) M1-M12 and measured WBC counts for six patients treated with D135. All but one cycle were used for personalisation and the last cycle for cross-validation. For patients (a)-(e) the PMs can predict the WBC count decrease after Ara-C administration in the last cycle where models M10 and M12 have a slower WBC recovery than M5. For patient (f) the WBC recovery from the PMs starts to early compared to the measured WBC counts.
