# Supplementary material for: Mathematical models for cytarabine-derived myelosuppression in acute myeloid leukaemia
Source: PLoS One. 2019 Jul 1;14(7):e0204540. doi: 10.1371/journal.pone.0204540 (PMC6602180; doi:10.1371/journal.pone.0204540)
Supplement: S3 Fig — The PMs provide good predictions for patient (a) and (f) but show mismatches in recovery times and nadir values for patients (b)-(e). (PDF) [file pone.0204540.s012.pdf]

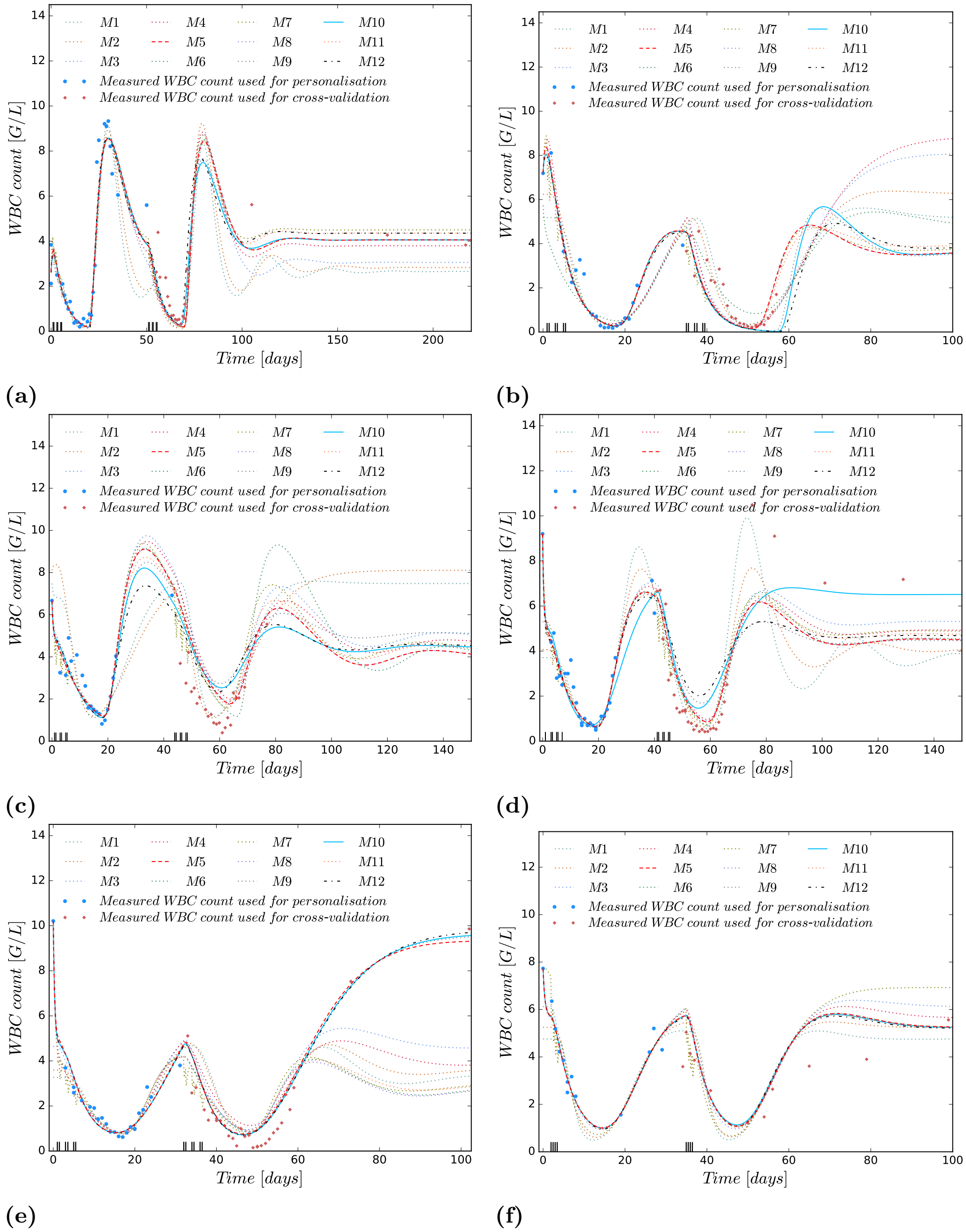

S3 Fig. Cross-validation of predicted white blood cell (WBC) counts from personalised models (PMs) M1-M12 and measured WBC counts for five patients (a)-(e) treated with d135 and one patient (f) treated with D12. The PMs provide good predictions for patient (a) and (f) but show mismatches in recovery times and nadir values for patients (b)-(e).
