# Supplementary material for: Mathematical models for cytarabine-derived myelosuppression in acute myeloid leukaemia
Source: PLoS One. 2019 Jul 1;14(7):e0204540. doi: 10.1371/journal.pone.0204540 (PMC6602180; doi:10.1371/journal.pone.0204540)
Supplement: S4 Fig — (PDF) [file pone.0204540.s013.pdf]

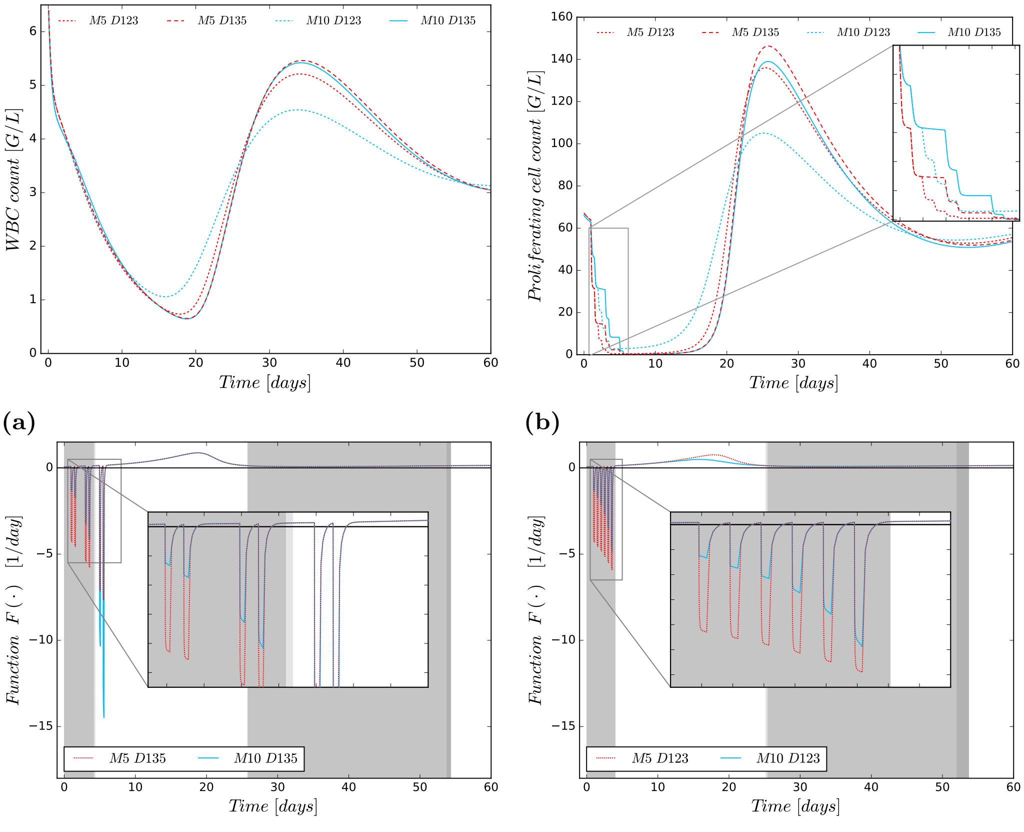

S4 Fig. Comparing personalised mathematical models (PMs) M5 and M10 for D123 and D135 schedules (exemplary patient I). For an exemplary patient, defined as I, the personalisation results in different model parameter values for M1–M12. For M5 and M10 shown above, the PMs are characterised by the following estimated model parameters. **(a)** Exemplary prediction of D123 and D135 schedules based on the gold-standard M5 and the promising extension M10. The D135 predictions are similar, the D123 predictions differ. **(b)** As (a), but proliferating cells x_{pr} are shown. The proliferating cell count of the PM based on M10 D123 recovers fastest, followed by M5 D123. For both D135 schedules the recovery takes longer. **(c)** Proliferation rate F for (a). Grey indicates at what times the WBC count is above the baseline WBC count ($x_{\text{ma}} \geq B$), resulting in reduced feedback. Compared to M5, the death rate for M10 is decreased at days 1 and 3, and increased at day 5. **(d)** As in (c), but for the D123 schedules. Compared to M5, the death rate for M10 is decreased at days 1,2, and 3. This explains why M10 resulted in a faster WBC recovery compared to M5. S5 Fig shows another example.

**Model**	B	k_{tr}	γ	slope	$x_{\text{pr}}(0)$	$x_{\text{tr}}(0)$	$x_{\text{ma}}(0)$
M5	3.32589	0.124924	1.19429	13.0743	67.1581	86.1515	6.63146
M10	3.33717	0.129158	1.16475	13.6057	66.0178	79.3855	6.66219

The values are quite close to another. Hence, the differences in S4 Figa are mainly due to different modelling assumptions. For the assumed impact of Ara-C concentration on the proliferation rate exponent in M10, the death rate F is reduced on days 1 and 3, S4 Figc. At day 5, Ara-C is administered when $x_{\text{ma}} < B$, indicated by a white background in S4 Figc. This leads to a higher absolute value of the feedback term and hence to an increased death rate compared to M5. The increased death rate at day 5 compensates the decreased ones at day 1 and 3, leading to almost identical dynamics for M5 D135 and M10 D135 in S4 Figa. For the D123 schedule, S4 Figd shows the reduced death rate F on all (grey) treatment days 1,2 and 3. As a result, the PM based on M10 recovers faster than the PM based on M5.
