# Supplementary material for: Mathematical models for cytarabine-derived myelosuppression in acute myeloid leukaemia
Source: PLoS One. 2019 Jul 1;14(7):e0204540. doi: 10.1371/journal.pone.0204540 (PMC6602180; doi:10.1371/journal.pone.0204540)
Supplement: S5 Fig — (PDF) [file pone.0204540.s014.pdf]

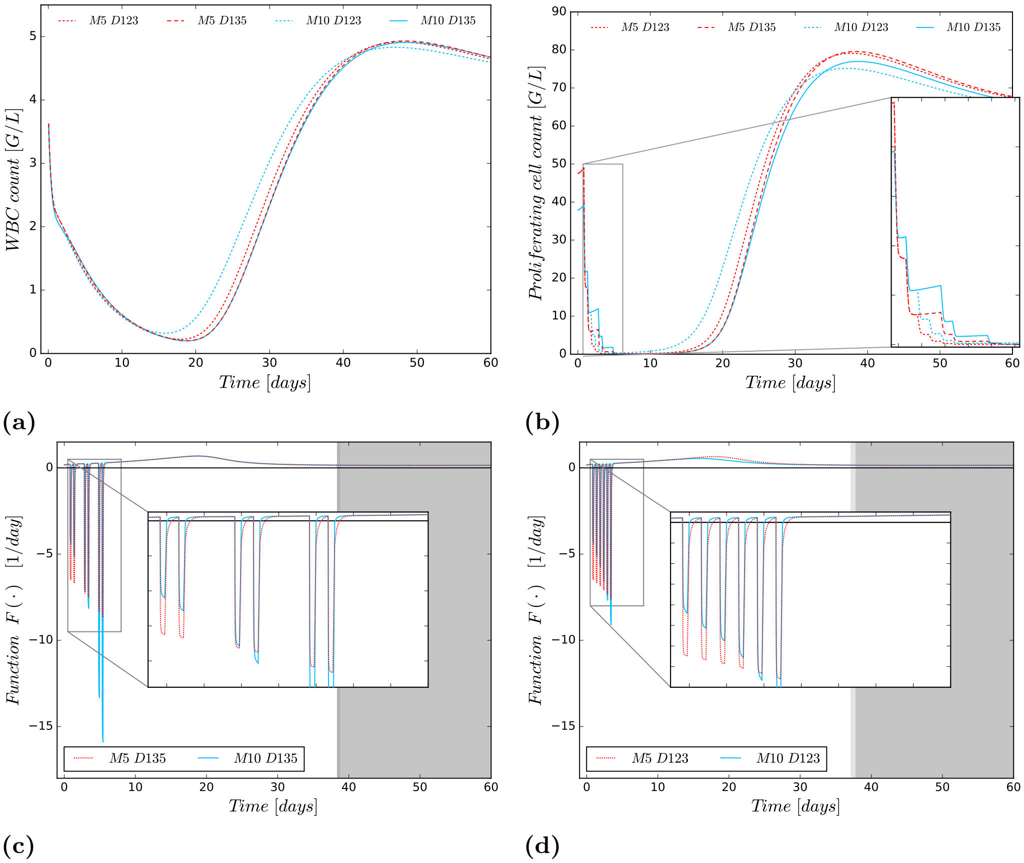

S5 Fig. Comparing personalised mathematical models (PMs) M5 and M10 for D123 and D135 schedules (exemplary patient II).
S5 Fig is organised as S4 Fig, but the PMs were calculated for a different set of white blood cell (WBC) counts from an exemplary patient defined as II. Here, the initial WBC count $x_{\mathrm{ma}}(t_0)$ is below the baseline WBC count value B (hence and in contrast to S4 Figc–d we start in a white, not grey, area). Thus, for identical model parameters the death rates of M10 would be increased for the D135 treatment and no compensation as in S4 Fig would occur. The estimated model parameters are:

Model	B	k_{tr}	γ	slope	$x_{\mathrm{pr}}(0)$	$x_{\mathrm{tr}}(0)$	$x_{\mathrm{ma}}(0)$
M5	4.33160	0.156974	0.482914	7.85156	47.5211	29.6839	3.83573
M10	4.35574	0.160689	0.471371	1.61711	37.9152	28.4292	3.81437

Obviously, the model parameter slope is reduced significantly for M10. As can be seen in S5 Figc this leads to a similar situation as in S4 Figc, with a reduced death rate on day 1, and an increased death rate on days 3 and 5. As a result, M5 D135 and M10 D135 in S5 Figa are almost identical.
The death rates for the D123 schedule are reduced compared to the D135 schedule, as shown in S5 Figc–d. As in S4 Figc–d, the reduction is more significant for the M10 model, which leads to the faster recovery of proliferating cells in S5 Figb and hence faster recovery of WBC counts in S5 Figa. S4 and S5 Figs show the two possible scenarios for the start of a chemotherapy, either $x_{\mathrm{ma}}(t_0) \geq B$ or $x_{\mathrm{ma}}(t_0) < B$. In both the WBC counts decrease after day 1 of the treatment. This decrease leads to increased feedback terms $(B/x_{\mathrm{ma}})^{\gamma}$ and hence to increased death rates. Therefore, administering Ara-C on days 1, 3, 5 instead of days 1, 2, 3 kills more healthy progenitor WBC. In M10 this important effect is stronger than in the gold-standard model M5.
