# Supplementary material for: Mathematical models for cytarabine-derived myelosuppression in acute myeloid leukaemia
Source: PLoS One. 2019 Jul 1;14(7):e0204540. doi: 10.1371/journal.pone.0204540 (PMC6602180; doi:10.1371/journal.pone.0204540)
Supplement: S6 Fig — (PDF) [file pone.0204540.s015.pdf]

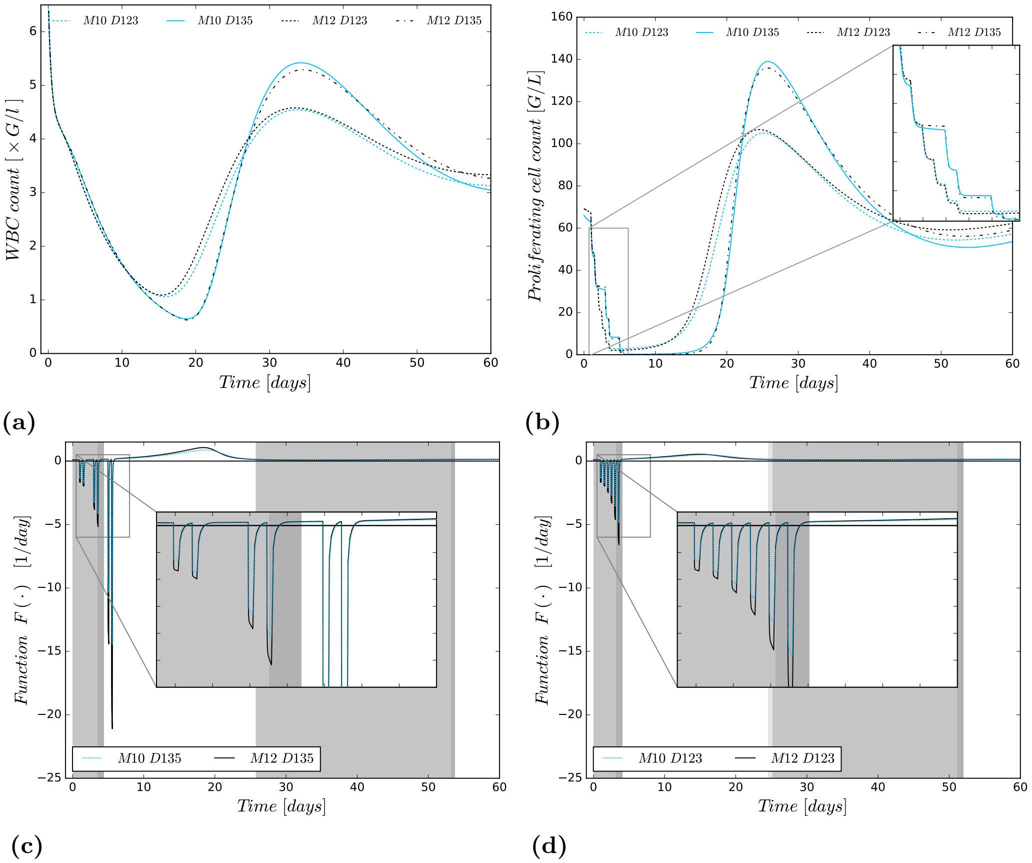

S6 Fig. Comparing personalised mathematical models (PMs) M10 and M12 for D123 and D135 schedules (exemplary patient I).

S6 Fig is organised as S4 Fig and based on the same set of white blood cell (WBC) counts, but comparing M10 and M12. The estimated model parameters are

**Model**	B	k_{tr}	γ	slope	$x_{\text{pr}}(0)$	$x_{\text{tr}}(0)$	$x_{\text{ma}}(0)$
M5	3.32589	0.124924	1.19429	13.0743	67.1581	86.1515	6.63146
M10	3.33717	0.129158	1.16475	13.6057	66.0178	79.3855	6.66219
M12	3.50825	0.127320	1.22611	10.0560	69.1946	80.0315	6.64934

Model M10 assumes a feedback term $(B/x_{\text{ma}})^{\gamma S(x_1)}$ for the proliferation rate F, while M12 assumes $(B_{\text{bm}}/(0.01 * x_{\text{pr}} + 0.99 * x_{\text{tr}}))^{\gamma S(x_1)}$. Administration of Ara-C leads to a reduction of WBC progenitor cells, compare S6 Figb. With a time delay of a few days this reduction then leads to a reduction of WBC counts as well, S6 Figa. Using WBC progenitor cells in the feedback term in M12 thus magnifies the effect described in S4 and S5 Figs. For the D135 schedule this is compensated by a reduced estimated slope value. For D123, the death rates of M12 are increased in the peaks, but the proliferation rate is slightly higher. As can be seen in S6 Figb, the M12 WBC progenitor cells recover faster, although they are below the M10 cell count at day 5.
