# Supplementary material for: Mathematical models for cytarabine-derived myelosuppression in acute myeloid leukaemia
Source: PLoS One. 2019 Jul 1;14(7):e0204540. doi: 10.1371/journal.pone.0204540 (PMC6602180; doi:10.1371/journal.pone.0204540)
Supplement: S7 Fig — Blue circles are the measured WBC counts of 23 AML patients described in section Clinical Data & Personalisation. One measurement was taken at timepoint 88.98 [days] with the value 7.18 [G/L] which is not shown in the VPCs. Red lines show the median (solid) and 5th and 95th percentiles (dashed) of measurements. The shaded areas represent the 95% confidence intervals around the 5th (blue), 50th (red) and 95th (blue) simulated percentiles of the model predictions. Regarding the VPCs, model M3 and M10 have an almost equivalent prediction accuracy. The 50% percentiles of measurements and model predictions perfectly overlap, thus supporting our individually based results from Table 3. The same applies to the start of the 5% and 95% percentiles until the nadir. After the nadir the 5% and 95% percentiles of the model predictions recover slightly faster/slower compared to the measurements. At day 30 the percentiles of measurements and model predictions coincide again. (PDF) [file pone.0204540.s016.pdf]

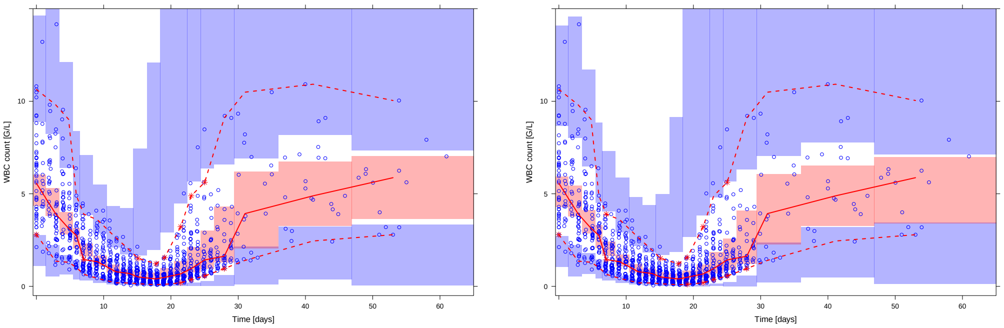

(a) **(b)**

S7 Fig. Visual predictive checks (VPCs), derived by 1000 simulations, for leukocytes [G/L] versus time [$days$] starting with the first measurement before dosing for model M3 (a) and M10 (with I1) (b). Blue circles are the measured WBC counts of 23 AML patients described in section *Clinical Data & Personalisation*. One measurement was taken at timepoint 88.98 [days] with the value 7.18 [G/L] which is not shown in the VPCs. Red lines show the median (solid) and 5th and 95th percentiles (dashed) of measurements. The shaded areas represent the 95 % confidence intervals around the 5th (blue), 50th (red) and 95th (blue) simulated percentiles of the model predictions. Regarding the VPCs, model M3 and M10 have an almost equivalent prediction accuracy. The 50 % percentiles of measurements and model predictions perfectly overlap, thus supporting our individually based results from Table 3. The same applies to the start of the 5 % and 95 % percentiles until the nadir. After the nadir the 5 % and 95 % percentiles of the model predictions recover slightly faster/slower compared to the measurements. At day 30 the percentiles of measurements and model predictions coincide again.
