# Supplementary material for: Mathematical models for cytarabine-derived myelosuppression in acute myeloid leukaemia
Source: PLoS One. 2019 Jul 1;14(7):e0204540. doi: 10.1371/journal.pone.0204540 (PMC6602180; doi:10.1371/journal.pone.0204540)
Supplement: S8 Fig — (a) As Fig 2, but with 500 simulations of our fitted two-compartment PK model with IIV on the clearance and the central volume. (b) Recovery times (trec) from 500 simulations each of models M3 and M10 (with I1) applying schedules D123 and D135 with inter-individual variability given as coefficient of variation (CV) on PK parameters clearance (45%) and central volume (70%). Red lines within the boxes are the medians, the upper and lower box limits are the first (Q1) and third quartiles (Q3) of the data. The lower whiskers will extend to the first trec values greater than the first quartiles minus the 1.5-times the interquartile ranges (IQR) (Q1 − 1.5 * IQR). Equivalently, the upper whiskers will extend to last trec values less than Q3 + 1.5 * IQR. Beyond the whiskers, data are considered as outliers and are plotted as individual points (+). The simulation study revealed that model M10 was more sensitive to different high-dose Ara-C treatment schedules compared to model M3 despite the high inter-individual PK variability. (PDF) [file pone.0204540.s017.pdf]

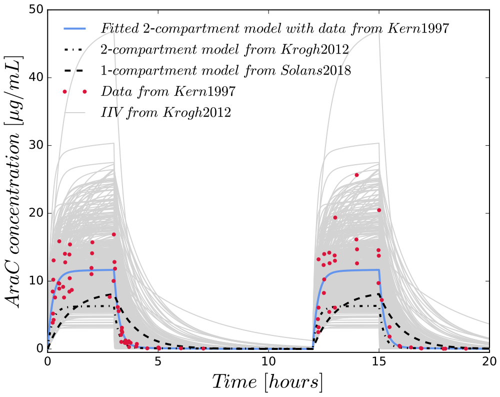

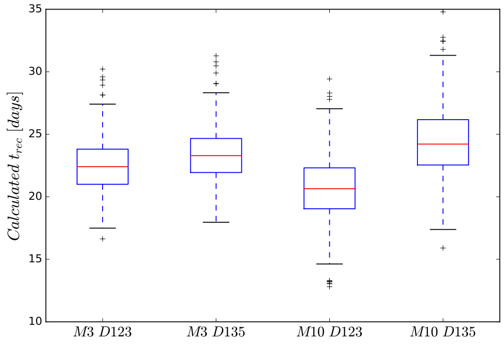

(b)

(a)

S8 Fig. Simulation study analysing the sensitivity of model M5 and M10 on inter-individual PK variability (IIV) when schedules D123 and D135 are applied.

(a) As Fig 2, but with 500 simulations of our fitted two-compartment PK model with IIV on the clearance and the central volume.

(b) Recovery times (t_{rec}) from 500 simulations each of models M3 and M10 (with I1) applying schedules D123 and D135 with inter-individual variability given as coefficient of variation (CV) on PK parameters clearance (45 %) and central volume (70 %). Red lines within the boxes are the medians, the upper and lower box limits are the first (Q1) and third quartiles (Q3) of the data. The lower whiskers will extend to the first t_{rec} values greater than the first quartiles minus the 1.5-times the interquartile ranges (IQR) ($Q1 - 1.5 * IQR$). Equivalently, the upper whiskers will extend to last t_{rec} values less than $Q3 + 1.5 * IQR$. Beyond the whiskers, data are considered as outliers and are plotted as individual points (+). The simulation study revealed that model M10 was more sensitive to different high-dose Ara-C treatment schedules compared to model M3 despite the high inter-individual PK variability.
